# Meeting Abstracts from the British Society of Echocardiography Annual Meeting: BSEcho 2022

**DOI:** 10.1186/s44156-023-00030-z

**Published:** 2023-09-28

## BSEcho 2022 Conference Report

Following two-years of being held virtually, it was a pleasure to welcome delegates to the first ever hybrid annual conference of the British Society of Echocardiography (BSE) in October 2022. This was held at the prestigious Queen Elizabeth II Conference Centre in the City of London, with fabulous views directly out over Westminster Cathedral and the Houses of Parliament.

After virtual conferences enabled two consecutive years of recording breaking attendance, it was a pleasure to welcome a total of 1300 attendees, including nearly 600 in person and 24 exhibitors and representatives from all the major echocardiography companies.

Lectures were spread over three auditoria during the conference with parallel sessions providing plenty of options for all delegates, including a stream dedicated entirely to trainee echocardiographers. In addition, there were live in-person sessions during the breaks providing delegates with the opportunity to gain insight from leading researchers in the field of echocardiography.

We were delighted to welcome Professor Otto Smiseth as our international guest speaker for BSEcho 2022. Professor Smiseth is a world-leading expert in the imaging of heart failure and provided an outstanding lecture on current trends and emerging techniques for the assessment of patients in heart failure with preserved ejection fraction. The Investigator of the Year award for 2022 was won by Dr James Willis for his investigation: *The right assessment: Utilising novel atrial markers to support pulmonary hypertension investigation*.

Prior to the meeting all members of the British Society of Echocardiographers were invited to submit abstracts which were then scored and shortlisted by an expert panel for originality, overall quality, significance and level of interest. Our thanks to the members of the panel: Dr Maria Paton; Dr Dave Oxborough; Dr Liam Ring; Dr Aimee Drane and Dr James Malcolmson for all their work in reviewing the abstracts.

The 2023 conference will take place on the 13th and 14th of October in Cardiff, at the International Conference Centre Wales. We are delighted to welcome Professor Judy Hung, director of echocardiography at Massachusetts General Hospital in Boston, and Professor Sanjay Sharma, professor of cardiology at St Georges Hospital in London, as our international and invited speakers. We look forward to seeing you there!

## ABS001 The positive predictive value of a family history of hypertrophic cardiomyopathy, dilated cardiomyopathy, bicuspid aortic valve or aortopathy for receiving a positive echocardiogram report in a UK district hospital

### Elise Robinson^1,2^, Jane Allen^1^, Rachel Richardson^1^

#### ^1^York and Scarborough Teaching Hospitals NHS Foundation Trust, Wiggington Road, York, UK; ^2^Newcastle University, Newcastle-Upon-Tyne, UK

*Echo Research & Practice* 2023, **10(Suppl 1)**:ABS001

**Background:** Heritable cardiac diseases cause significant morbidity and mortality. A Service Evaluation of the York Hospital screening program, which utilises echocardiography for diagnosis, was undertaken. The Positive Predicative Value (PPV) of a family history (FH) for heritable cardiac disease was investigated to evaluate the screening program.

**Methods:** Records of patients referred to the Cardio-Respiratory department for a FH of Bicuspid Aortic Valve (BAV), Aortopathy, Hypertrophic Cardiomyopathy (HCM) or Dilated Cardiomyopathy (DCM) between 2010 and 2021 were examined. Patients with a positive echocardiogram report for the pathology being screened for were classed as True Positives. This allowed the PPV for FH of each condition to be calculated.

**Results:** PPV for FH of DCM, BAV and HCM were low (0%, 3% (95% CI [0–11.5]) and 6% (95% CI [2–10]) respectively). Aortopathy had a higher PPV overall at 22% (95% CI [8.6–35.8]). Ages of the True Positives were significantly higher than the False Positives for both aortopathy and HCM (p = 0.021, p = 0.0079). Comparing disease prevalence in the study population to the general population showed that BAV prevalence was not significantly different (p = 0.52) whereas aortopathy and HCM prevalence’s were significantly higher than the general population (p = 0.0002, 0.00001). Male gender (p = 0.0101), 1st degree proband (p = 0.0101) and being > 45 years old (p = 0.002) were significantly associated with the True Positive aortopathy group and being > 45 years old was significantly associated with True Positive HCM group (p = 0.029).

**Conclusion:** Analysis of the BAV and DCM data is limited by sample size. For HCM and aortopathy this data supports that initiating screening later in life could improve the efficacy of screening. The characteristics found to be significantly associated with True Positives could inform a prospective study.

## ABS002 Normal right ventricular augmentation during stress echocardiography—a comparative study

### Sahar Alborikan^1,2,3^, Bejal Pandya^4^, Katherine von Klemperer^4^, Sveeta Badiani^1,2^, Aisha Althunayyan^1^, Sanjeev Bhattacharyya^1,5^, Jet van Zalen^6^, Guy Lloyd^1,5^

#### ^1^Barts Heart Centre, St Bartholomew's Hospital, Barts Health NHS Trust, London, UK; ^2^William Harvey Research Institute, Queen Mary University of London, London, UK; ^3^King Fahad Specialist Hospital, Cardiac Department, Dammam, Saudi Arabia; ^4^Grown-up Congenital Heart Disease Services, Barts Heart Centre, St Bartholomew's Hospital, Barts Health NHS Trust, London, UK;^5^Institute of Cardiovascular Science, University College London, London, UK; ^6^Department of Cardiology, Eastbourne District General Hospital, East Sussex NHS Trust, Eastbourne, UK

*Echo Research & Practice* 2023, **10 (Suppl 1)**:ABS002

**Background:** The assessment of right ventricular (RV) contractile reserve (CR) during exercise echocardiography in healthy subjects is not well described. We aimed to describe normal RV CR in healthy cases and to compare CR to patients with defined RV compromise because of previous repair of Tetralogy of Fallot.

**Methods:** 40 healthy individuals with satisfactory RV windows were randomly selected and retrospectively analysed from previously published study (marathon study). These cases were compared to 100 adult patients with repaired TOF. We defined RV CR by the change in tricuspid lateral annular systolic velocity (ΔRVS’), change in tricuspid annular plane systolic excursion (ΔTAPSE), and change in fractional area change (ΔFAC). All parameters were evaluated at baseline and at peak stress.

**Results:** During exercise, RVS’ was increased by 60 ± 20%, followed by TAPSE 48 ± 15%, and the lowest with FAC by 32 ± 10%. These ranges were significantly higher than in TOF population. RV CR was greater in males than females for all RV functional measures (34 ± 10 vs 28 ± 10, %, p < 0.05); (50 ± 10 vs 44 ± 11, %, p < 0.05); (61 ± 12 vs 52 ± 11, %, p < 0.05), for ΔFAC, ΔTAPSE, and ΔRVS’, respectively. There was no association between RV CR and functional capacity parameters in healthy individuals, whereas in patients with repaired TOF there was a significant association with peak absolute VO_2_ (ml/min) (r = 0.36, p < 0.001, with ΔFAC).

**Conclusion:** We have presented normal values for RV CR parameters in healthy cases during stress echocardiography. RV CR is an important determinant of exercise capacity in patients with abnormal RV function but not in normal subjects.

## ABS003 Reproducibility and repeatability of biventricular function/volume and strain parameters by 2D and 4D stress echocardiography in adult patients with repaired TOF

### Sahar Alborikan^1,2,3^, Bejal Pandya^4^, Katherine von Klemperer^4^, Sveeta Badiani^1,2^, Reuben Dane^1^, Delfin Encarnacion^1^, Roma Amor Bingcang^1^, Ricardo Prista Monteiro^1,5^, Sanjeev Bhattacharyya^1,6^, Guy Lloyd^1,6^

#### ^1^Barts Heart Centre, St Bartholomew's Hospital, Barts Health NHS Trust, London, UK; ^2^William Harvey Research Institute, Queen Mary University of London, London, UK; ^3^King Fahad Specialist Hospital, Cardiac Department, Dammam, Saudi Arabia; ^4^Grown-up Congenital Heart Disease Services, Barts Heart Centre, St Bartholomew's Hospital, Barts Health NHS Trust, London, UK; ^5^Faculty of Medicine and Biomedical Sciences, University of Algarve, Faro, Portugal; ^6^Institute of Cardiovascular Science, University College London, London, UK

*Echo Research & Practice* 2023, **10 (Suppl 1)**:ABS003

**Background:** The use of 2D and 4D during stress echocardiography to undertake complex measures in complex patients like patients with repaired TOF is challenging and the validity of these measures is not known yet.

**Methods:** For test–retest variability, 20 patients with repaired TOF with no or mild pulmonary regurgitation were selected randomly and underwent a cardiopulmonary exercise test (CPET) with echocardiography. Intra-observer variability study was performed for all 20 patients by the same observer. Interobserver variability study was performed for 5 patients by different experienced observer. Intraclass correlation coefficient (ICC), and coefficients of variation (COV) were used to quantify reproducibility and variability.

**Results:** For 2D measures, better reproducibility was observed for semiautomated 2D strain measures than 2D functional measures for biventricular systolic function at baseline and during the stress (ICC > 0.90 vs > 0.70, p < 0.001), with least COV was observed (COV < 10%). 4D semiautomated volumetric measures demonstrated less reproducibility during stress with highest COV was observed for 4D RV volume parameters (COV, 35%), followed by 4D LV volume parameters (COV, 27%). CPET had an excellent agreement of all measures (ICC > 0.90) with very low COV (< 10%).

**Conclusion:** Semiautomated echo measures outperformed manual measures during stress echocardiography and can be performed with acceptable reproducibility. Variability is at highest for 4D semiautomated measures despite good reproducibility, while lowest variability was observed for 2D semiautomated measures of myocardial deformation.

## ABS004 Right and left ventricular structural, functional characteristics, volumes, mechanics and myocardial augmentation during exercise; how do they predict exercise capacity in patients with Tetralogy of Fallot and pulmonary regurgitation

### Sahar Alborikan^1,2,3^, Bejal Pandya^4^, Katherine von Klemperer^4^, Sveeta Badiani^1,2^, Reuben Dane^1^, Delfin Encarnacion^1^, Roma Amor Bingcang^1^, Ricardo Prista Monteiro^1,5^, Sanjeev Bhattacharyya^1,6^, Guy Lloyd^1,6^

#### ^1^Barts Heart Centre, St Bartholomew's Hospital, Barts Health NHS Trust, London, UK; ^2^William Harvey Research Institute, Queen Mary University of London, London, UK; ^3^King Fahad Specialist Hospital, Cardiac Department, Dammam, Saudi Arabia; ^4^Grown-up Congenital Heart Disease Services, Barts Heart Centre, St Bartholomew's Hospital, Barts Health NHS Trust, London, UK; ^5^Faculty of Medicine and Biomedical Sciences, University of Algarve, Faro, Portugal; ^6^Institute of Cardiovascular Science, University College London, London, UK

*Echo Research & Practice* 2023, **10 (Suppl 1)**:ABS004

**Background:** Identifying exercise determinants in patients with repaired Tetralogy of Fallot (rTOF) is complex. We sought to investigate exercise performance and to identify the best exercise predictors.

**Methods:** We prospectively recruited 100 patients with rTOF, 60 patients with severe PR (SPR), and 40 patients with no PR (controls). All patients underwent cardiopulmonary exercise testing with echocardiography. Right ventricle (RV) contractile reserve (CR) was defined by the change in peak systolic velocity (ΔRVS’), and change in fractional area change (ΔFAC). Left ventricle (LV) CR was defined by the change in systolic function (ΔLVS’), and change in global longitudinal strain (ΔLVGLS).

**Results:** There was no significant difference in the reduced exercise performance between the SPR and control groups by peak absolute oxygen consumption VO2(1695 ± 627 vs 1744 ± 521, ml/min, p > 0.05). During exercise, lower RV CR was observed in the SPR group by ΔRVS’ (41 ± 28 vs 48 ± 20%, p < 0.05); and ΔFAC (20 ± 15 vs 23 ± 16,%, p < 0.05), while it was greater for LV CR by ΔLVS’(67 ± 34 vs 61 ± 28%, p < 0.05). Change in ΔLVGLS was the same (15 ± 17 vs 16 ± 15,%, p > 0.05). There were no associations between exercise measures with the degree of PR and RV volumes at rest and during exercise. Augmentation of LVGLS and FAC were shown independent associations with peak VO2(r = 0.55, r = 0.45, p < 0.05).

**Conclusion:** there was an overall marked reduction in exercise capacity but not a difference between those with and without PR. The degree of exercise limitations is more dependent upon the ability of RV and LV to augment longitudinal function rather than to severity of PR.

## ABS005 Complex myocardial mechanics in adult patients with repaired TOF—a novel study assessing myocardial work and mechanical dispersion at rest and during exercise

### Sahar Alborikan^1,2,3^, Bejal Pandya^4^, Katherine von Klemperer^4^, Sveeta Badiani^1,2^, Reuben Dane^1^, Delfin Encarnacion^1^, Roma Amor Bingcang^1^, Ricardo Prista Monteiro^1,5^, Sanjeev Bhattacharyya^1,6^, Guy Lloyd^1,6^

#### ^1^Barts Heart Centre, St Bartholomew's Hospital, Barts Health NHS Trust, London, UK; ^2^William Harvey Research Institute, Queen Mary University of London, London, UK; ^3^King Fahad Specialist Hospital, Cardiac Department, Dammam, Saudi Arabia; ^4^Grown-up Congenital Heart Disease Services, Barts Heart Centre, St Bartholomew's Hospital, Barts Health NHS Trust, London, UK; ^5^Faculty of Medicine and Biomedical Sciences, University of Algarve, Faro, Portugal; ^6^Institute of Cardiovascular Science, University College London, London, UK

*Echo Research & Practice* 2023, **10 (Suppl 1)**:ABS005

**Background**: Assessment of LV myocardial work (MW) and biventricular mechanical dispersion (MD) in patient with repaired TOF (rTOF) is novel. We sought to investigate the LV MW and biventricular MD response during stress echocardiography.

**Methods:** We analysed MW and MD at baseline and at low exercise intensity for 100 adult patients with repaired TOF, 60 patients with severe pulmonary regurgitation (SPR), and 40 patients with negligible PR (NPR). MW was derived as the area of pressure strain loop using speckle tracking echocardiography and blood pressure. MD was derived as the SD of time Q/R wave on ECG to peak longitudinal strain and expressed in millisecond.

**Results:** Reduced MW indices were observed in the entire population with mean global work efficiency (MWE) was 85 ± 7%. Overall mean of global work index (GWI) was 1198 ± 312 mmHg%, 1701 ± 303 mmHg% for global constructive work (GCW), and 293 ± 194 mmHg% for global wasted work (GWW). The SPR group had lower MWE, lower GCW and higher GWW. During exercise, overall ΔMWE decreased by − 2 ± 10%, ΔGWI increased by 36 ± 43%, ΔGCW increased by 68 ± 40%, and ΔGWW increased by 120 ± 110%. Overall resting mean value of RVMD was 46 ± 18,ms, while 64 ± 11,ms for LVMD, and higher values were observed during exercise. Changes in MWE, MWW, and LV and RVMD were closely associated with peak oxygen uptake (r = 0.33, r = 0.41, r = 0.36, r = 0.47, p < 0.001).

**Conclusions**: LV MW indices are reduced and biventricular MD are pronounced in rTOF patients. Augmentation of MW parameters and timing were associated with objective exercise ability, suggesting that they are potential determinants of cardiopulmonary capability.

**Figure 1 (abstract ABS005):**
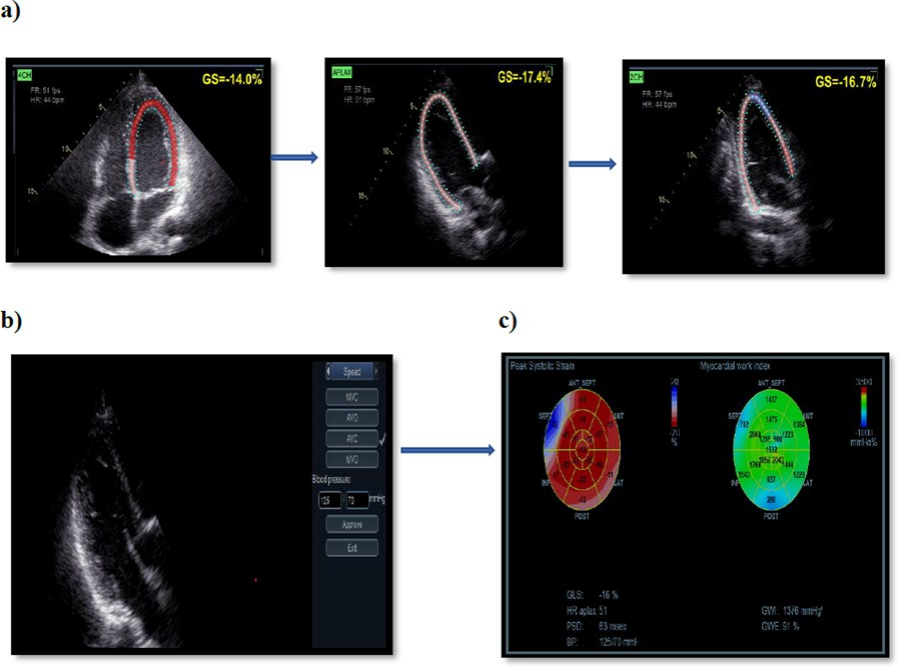
Determination of LV myocardial work. a) the process of measuring GLS from apical 4,3 and 2 chambers. b) systolic and diastolic blood pressure levels have to be entered for LVMW assessment. c) LVMW end results

## ABS006 Systo-diastolic coupling and prognostic impact of early systolic dysfunction in HFpEF

### Haotian Gu^1^, Jessica Webb^2^, Luca Faconti^1^, Reza Razavi^3^, Ajay M Shah^1^, Gerry Carr-White^2^, Phil Chowienczyk^1^

#### ^1^King’s College London, British Heart Foundation Centre, London, UK: ^2^Cardiothoracic Centre, St Thomas’ Hospital, London, UK; ^3^Imaging Sciences and Biomedical Engineering, King’s College London, UK

*Echo Research & Practice* 2023, **10 (Suppl 1)**:ABS006

**Background:** Many patients with heart failure, particularly that associated with hypertension, have preserved ejection fraction (HFpEF) where impaired relaxation is thought to be the primary cardiac abnormality. However, early systolic dysfunction could also be an important feature of HFpEF.

**Aims:** We examined the relationship of early systolic function as measured by first-phase ejection fraction (EF1) to diastolic function and whether EF1 predicts adverse outcomes (a combined end-point of heart failure re-hospitalisation and all-cause mortality) in patients with HFpEF.

**Methods:** EF1 was measured in hypertensive subjects with no evidence of heart failure, but with varying degrees of diastolic dysfunction and in patients with HFpEF. The relationship of EF1 to diastolic function under resting conditions and after an acute intervention to reduce cardiac pre-load was examined.

**Results:** There was a progressive impairment of EF1 with degree of diastolic dysfunction (mean ± SD EF1:27.3 ± 4.4, 23.4 ± 9.1 and 17.9 ± 5.2% for no diastolic dysfunction, diastolic dysfunction and HFpEF respectively, p < 0.001). An acute reduction in cardiac pre-load resulted in an increase in EF1 which correlated with an improvement in E/e’ (p < 0.01). In 177 HFpEF patients followed for a median of 19.3 months, there were 40 deaths and 61 re-hospitalisations. EF1 was the most powerful predictor of events (hazard ratio for EF1 < 19.4% compared to ≥ 19.4% 2.750 [95%CI: 1.737–4.353], p < 0.001) and improved the C-statistic over a model incorporating age, BMI, stroke, COPD, end-diastolic volume, arterial and ventricular elastance (p < 0.001).

**Conclusion:** Early systolic dysfunction may be a major determinant of outcome in HFpEF, is at least partially reversible, and a potential target for therapy.

## ABS007 Early left ventricular systolic function is a more sensitive predictor of adverse events in heart transplant recipients

### Zhenxing Sun^1,2^*, Yu Cai^1,2^*, Yujia Yang^3^*, Lei Huang^1,2^, Yuji Xie^1,2^, Ziming Zhang^1,2^, Yuman Li^1,2^, Jing Wang^1,2^, Lingyun Fang^1,2^, Yali Wang^1,2^, Qing Lv^1,2^, Li Zhang^1,2^†, Mingxing Xie^1,2^†, Haotian Gu^3^†

#### ^1^Department of Ultrasound, Union Hospital, Tongji Medical College, Huazhong University of Science and Technology, Wuhan 430,022, China; ^2^Hubei Province Key Laboratory of Molecular Imaging, Wuhan 430,022, China; ^3^British Heart Foundation Centre of Research Excellence, King’s College London, UK

*Zhenxing Sun, Yu Cai and Yujia Yang are co-first authors

^†^Li Zhang, Mingxing Xie and Haotian Gu are Co-senior author

*Echo Research & Practice* 2023, **10 (Suppl 1)**:ABS007

**Background:** First-phase ejection fraction (EF1) is a novel measure of early systolic function. This study was to investigate the prognostic value of EF1 in heart transplant recipients.

**Methods:** Heart transplant recipients were prospectively recruited consecutively at the Union Hospital, Wuhan, China between January 2015 and December 2019. All patients underwent clinical examination, biochemistry measures [brain natriuretic peptide (BNP) and creatinine] and echocardiography. The primary endpoint was a combined events of all-cause mortality and graft rejection.

**Results:** In 277 patients (aged 48.6 + 12.5 years) followed for a median of 38.7 (interquartile range: 18.3) months, there were 35 (12.6%) patients had adverse events. EF1 was associated with BNP ((B  = − 0.220, p < 0.001) and was significantly lower in patients with events compared to those without. EF1 had the largest area under the curve in ROC analysis compared to other measures. A cut-off value of 25.8% for EF1 had a sensitivity of 96.3% and a specificity of 97.1% for prediction of events. EF1 was the most powerful predictor of events with hazard ratio per 1% change in EF1: 0.628 (95%CI: 0.555–0.710, p < 0.001) after adjustment for ejection fraction and global longitudinal strain.

**Conclusion:** Early systolic function as measured by EF1 is a powerful predictor of adverse outcomes after heart transplant. EF1 may be useful in risk stratification and management of heart transplant recipients.

## ABD008 Increased detection of pericardial effusion during the COVID-19 pandemic

### Amy Wilson^1^, Chris Ellis^1^, Eveline Lee^1^

#### ^1^Shrewsbury and Telford Hospital NHS Trust

*Echo Research & Practice* 2023, **10 (Suppl 1)**:ABS008

**Background:** Pericardial effusions (PE) occur when there is an excess of fluid accumulating within the pericardial space. We have observed an increase in the number of PE’s detected amongst all transthoracic echocardiography (TTE) scans performed since the start of the COVID-19 pandemic irrespective of cause for referral. This is interesting given that the most common cause of PE’s in the Western World is considered to be post-viral infection.

**Aims:** Validate a significant increase in the rate of PE detection via TTE from January 2020-December 2021 compared to the previous 3 years and compare PE detection with national COVID-19 infection data.

**Methods:** All TTE scans performed between January 2017 and December 2021 were utilised to generate rates of PE detection. A t-test was performed to assess for a significant difference in PE detection pre-COVID-19 (January 2017-December 2019) and during the pandemic (January 2020-December 2021). Data on the incidence of COVID-19 cases in the UK was gathered from the Gov.uk website.

**Results:** A total of 37,069 TTE’s were performed pre-COVID-19 and 24,125 scans post-COVID-19. Majority of the 2020–2021 TTE’s were performed in low risk COVID-19 patients. There were significantly more PE’s detected post-COVID-19 compared with pre-COVID-19 with rates of detection of 0.14 and 0.05 respectively (p < 0.001). Detection of PE’s increased from 2017–2021, despite a decrease in total scans performed post-COVID-19 (Figure 1). Comparison with national COVID-19 infection data shows a peak in PE incidence following a peak in infections (Figure 2).

**Conclusion:** We have noticed a significant increase in PE detection since the start of the COVID-19 pandemic. This appeared to track the incidence of national COVID-19 infections.

**Figure 1 (abstract ABS008):**
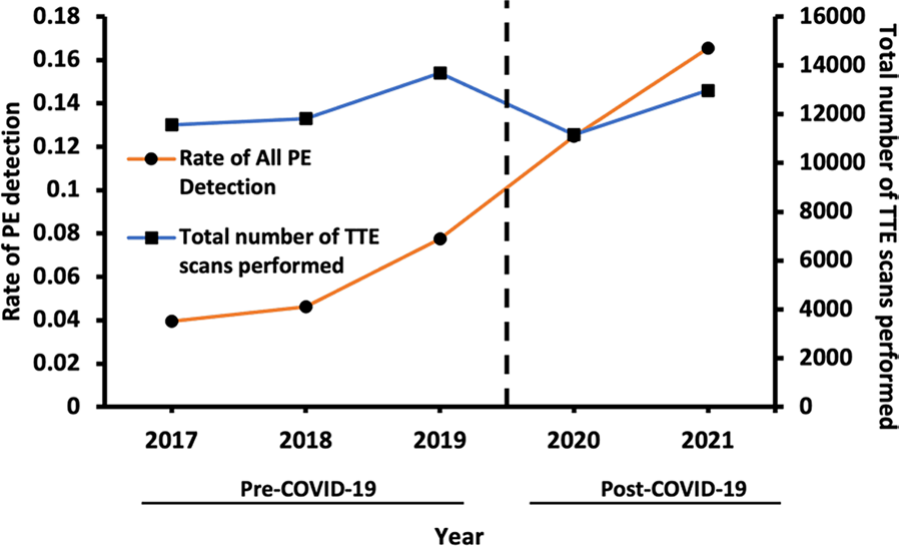
The rate of detection of PE’s via TTE has increased overall from 2017–2021, despite a decrease in total scans performed post-COVID-19. Rate of PE detection each year and total number of TTE’s performed. *Abbreviations: PE, pericardial effusion; TTE, transthoracic echocardiogram*

**Figure 2 (abstract ABS008):**
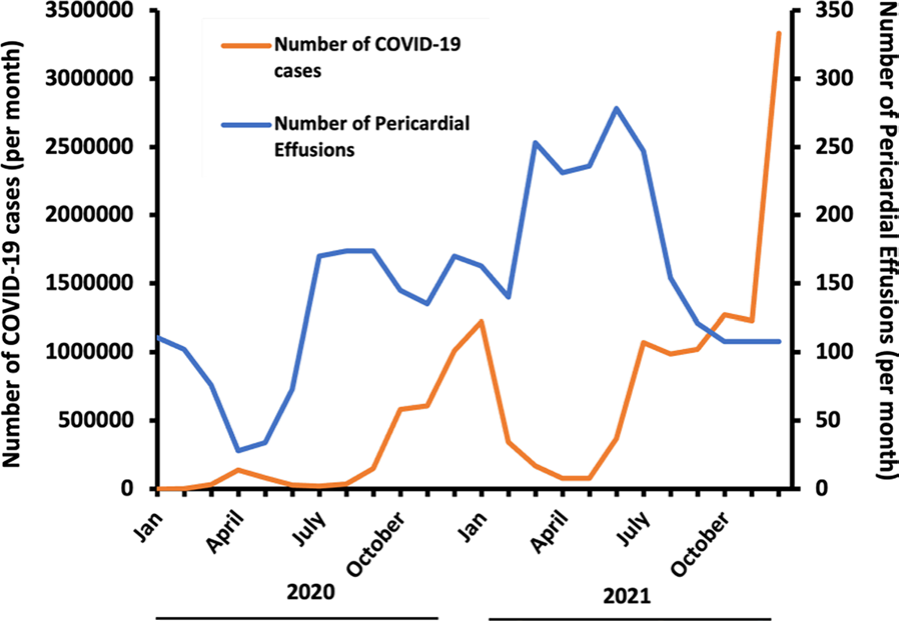
Peak in COVID-19 infections precedes a peak in pericardial effusion cases. January 2020-December 2021. COVID-19 data obtained from https://coronavirus.data.gov.uk/

## ABS009 Inter-technique agreement and test–retest reproducibility of assessing left atrial function: a comparison between transthoracic echocardiography and cardiovascular magnetic resonance imaging

### Aseel Alfuhied^1,2^, Gaurav S Gulsin^1^, Jian L Yeo^1^, Christopher D Steadman^3^, Anna-Marie Marsh^1^, Kelly Parke^1^, Jayanth Ranjit Arnold^1^, Gerry P McCann^1^, Anvesha Singh^1^

#### ^1^Department of Cardiovascular Sciences, University of Leicester and Cardiovascular Theme, National Institute for Health Research (NIHR) Leicester Biomedical Research Centre, Glenfield Hospital, Leicester, UK; ^2^College of Applied Medical Sciences, King Saud bin Abdulaziz University for Health Sciences, Riyadh, Kingdom of Saudi Arabia; ^3^Department of Cardiology, Poole Hospital NHS Foundation Trust, Poole, UK

*Echo Research & Practice* 2023, **10 (Suppl 1)**:ABS009

**Background:** Left atrial (LA) function is a novel cardiovascular imaging biomarker, but the agreement of different techniques and reproducibility is not known.

**Aims:** We aimed to investigate: (i) The inter-technique agreement and (ii) The test–retest reproducibility of both transthoracic echocardiography (TTE) and cardiac MRI (CMR) derived LA strain (LAS) and volumetric assessment in people with and without cardiovascular disease.

**Methods:** 192 participants were recruited: Asymptomatic Type 2 diabetes (T2D, n = 74), severe aortic stenosis (n = 65) and healthy volunteers (n = 53) had TTE and CMR on the same day for inter-technique agreement. Another ten participants with T2D had both scans repeated 11 ± 4 days later for test–retest reproducibility. TTE images were analysed using TomTec-ARENA (v2.4) and Medis Suite (v3.1) for CMR images. All analyses were performed by a single-blinded operator using identical techniques for TTE and CMR: LA volumes and emptying fraction (EF) were quantified using biplane area-length method. LAS corresponding to LA reservoir, conduit, and booster pump was calculated using the average of 4- and 2-chamber values.

**Results:** As shown in Table 1, TTE and CMR agreement was moderate (ICC 0.55–0.69) for LAEF and strain parameters. CMR reproducibility was good to excellent for LA volumes, EF, reservoir and booster pump-LAS, whilst on TTE LA volumes and conduit-LAS had good reproducibility (Table 2 & Figure 1).

**Conclusion:** There is a modest agreement between TTE and CMR for LA function assessment. Conduit-LAS is more reproducible on TTE. LAEF, reservoir and booster pump-LAS are more reproducible on CMR, suggesting the use of CMR in assessing LA function in longitudinal studies.

**Table 1 (abstract ABS009):** Inter-technique agreement of LA parameters CMR vs TTE (n = 192)

Parameter	CMR (Mean ± SD)	TTE (Mean ± SD)	p-value	Bias (Limits of agreement)	ICC (p-value)
LAV_Max_ (ml)	79.7 ± 24.8	49.2 ± 18.0	** < 0.001**	30.5 (-4.18, 65.2)	0.50 (< 0.001)
LAV_Min_ (ml)	38.5 ± 18.4	24.5 ± 13.3	** < 0.001**	14.0 (-7.46, 35.5)	0.72 (< 0.001)
LAEF (%)	53.0 ± 9.20	51.4 ± 9.84	**0.039**	1.58 (-19.1, 22.2)	0.55 (< 0.001)
LAS_r (%)	30.3 ± 8.79	31.2 ± 7.51	0.145	-0.89 (-17.45, 15.7)	0.63 (< 0.001)
LAS_cd (%)	15.6 ± 6.84	16.1 ± 6.45	0.321	-0.47(-13.1, 12.2)	0.69 (< 0.001)
LAS_bp (%)	15.0 ± 5.14	15.3 ± 5.0	0.458	-0.31 (-11.4, 10.7)	0.55 (< 0.001)

p-value by paired t-test

Abbreviations: LAVmax = Left atrial maximum volume, LAVmin = Left atrial minimum volume, LAEF = left atrial emptying fraction, LAS_r = Left atrial strain at reservoir phase, LAS_cd = Left atrial strain at conduit phase, LAS_bp = Left atrial strain at booster pump phase, ICC = intraclass correlation measured for absolute agreement

**Table 2 (abstract ABS009):** Test–retest reproducibility of LA parameters using CMR vs TTE (n = 10)

Parameter	CMR			TTE		
	Scan 1 (Mean ± SD)	Scan 2 (Mean ± SD)	ICC (p-value)	Scan 1 (Mean ± SD)	Scan 2 (Mean ± SD)	ICC (p-value)
**LAV_Max (ml)**	58.2 ± 18.8	53.8 ± 12.7	0.90 (0.001)	44.2 ± 14.7	41.3 ± 11.5	0.83 (0.007)
**LAV_Min (ml)**	28.7 ± 10.9	28.0 ± 7.2	0.92 (0.001)	18.0 ± 6.3	18.5 ± 6.1	0.88 (0.003)
**LAEF (%)**	51.1 ± 6.2	48.0 ± 5.6*	0.83 (0.002)	59.3 ± 4.75	55.6 ± 4.49	0.40 (0.174)
**LAS_r (%)**	29.2 ± 6.5	27.9 ± 7.8	0.83 (0.009)	33.8 ± 3.70	31.4 ± 6.76	0.35 (0.260)
**LAS_cd (%)**	15.1 ± 6.0	12.6 ± 5.3	0.73 (0.024)	16.7 ± 3.41	15.0 ± 4.16	0.80 (0.008)
**LAS_bp (%)**	14.0 ± 5.9	15.4 ± 6.0	0.78 (0.020)	17.1 ± 3.18	16.3 ± 4.41	0.23 (0.362)

*Scan 1 vs scan 2 by paired t-test analysis p-value < 0.05

Abbreviations as Table 1

**Figure 1 (abstract ABS009):**
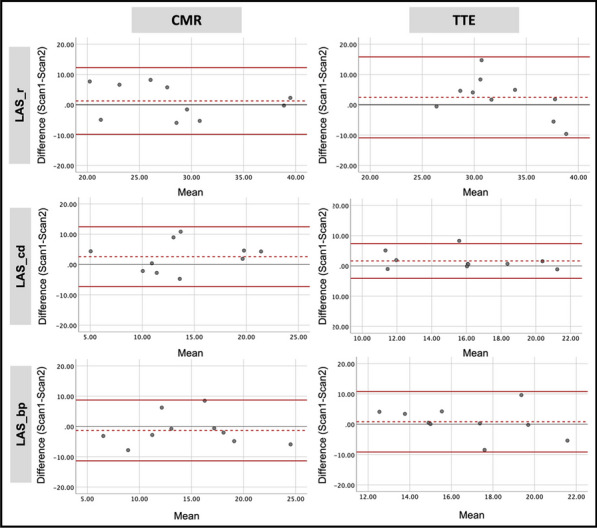
Bland–Altman plots for test–retest reproducibility of LA strain parameters using CMR and TTE

## ABS010 Aortic root and ascending aorta dilatation: a new pandemic?

### Rafia Begum^1^, Alexandra Thompson^2^

#### ^1^Echo Department, Freeman Hospital, Newcastle upon Tyne NHS Trust, Newcastle, UK; ^2^Royal Victoria Infirmary, Newcastle upon Tyne NHS Trust, Newcastle, UK

*Echo Research & Practice* 2023, **10 (Suppl 1)**:ABS010

**Background:** In 2020, the British Society of Echocardiography (BSE) produced updated normal reference ranges for the aortic root and ascending aorta. After implementation, clinicians at Newcastle upon Tyne NHS Foundation Trust noticed increasing numbers of reports with “aortic root dilatation” and were unsure how to proceed.

**Aims:** In March 2022 we conducted an audit to determine the numbers of patients with a report of a dilated aortic root/ascending aorta according to the new BSE criteria.

**Methods:** We selected 100 consecutive patients from the local echo database with height documented. Absolute and indexed measurements were noted for the aortic sinuses, sinotubular junction, and ascending aorta. Age, sex, valve morphology, family history of aortopathy, hypertension, and other cardiovascular risk factors were obtained from electronic records.

**Results:** 28% of patients had aortic root and/or ascending aorta dilatation (Figure 1). Mean age was 69 years and 61% were male. Within the cohort with dilatation, 25 had tricuspid aortic valves (3 others prosthetic valves), 64% had hypertension, and 56% had other cardiovascular risk factors. One had a family history of aortopathy. The vast majority of dilatation occurred in patients aged over 60 years and with an absolute aortic dimension < 40 mm (Figure 2).

**Conclusion:** NORRE reference intervals would allow for 2.5% of healthy people to have aortic roots/ascending aortas above the normal range. Patients are not normal by definition and may have predisposing conditions causing aortic dilatation. What requires surveillance in this setting is not clear, but a 28% surveillance rate is not sustainable for current services. A regional strategy to rationalise this is being ratified. A national response needs consideration.

## References

Harkness, A., Ring, L., Augustine, D., Oxborough, D., Robinson, S. and Sharma, V on behalf of the Education Committee of the British Society of Echocardiography (2020) Normal reference intervals for cardiac dimensions and function for use in echocardiographic practice: a guideline from the British Society of Echocardiography [pdf] Available at: https://echo.biomedcentral.com/track/pdf/10.1530/ERP-19-0050.pdf [Accessed 17th July 2022].

**Figure 1 (abstract ABS010):**
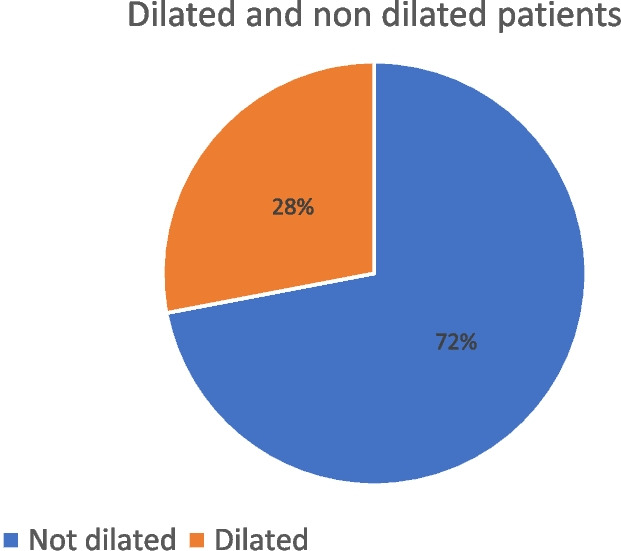
Pie chart presenting the percentage of dilated to non dilated aortic root patients

**Figure 2 (abstract ABS010):**
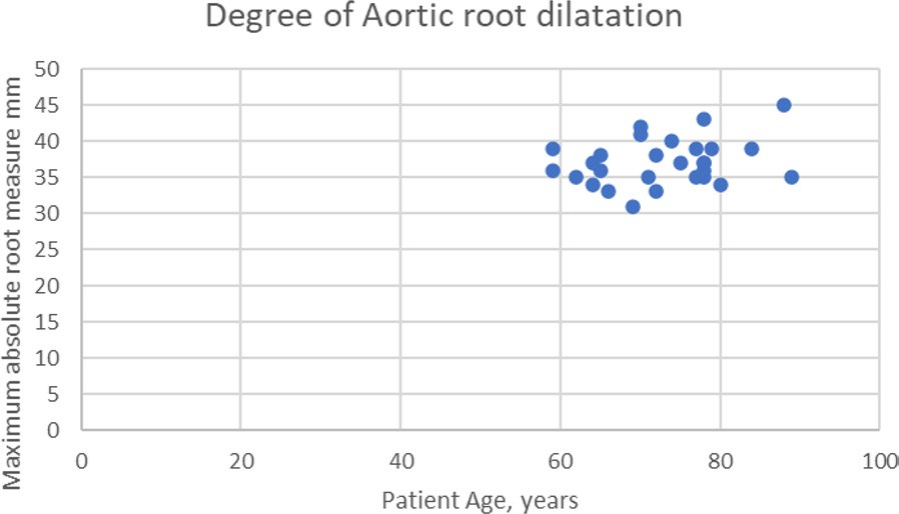
Plot of the 28 dilated patients by age against the maximum absolute aortic root measurement in millimetres

## ABS011 Inappropriate echo referrals from GPs to ‘assess left ventricular function’: Do we do what we think we do?

### Albert George^1^, Carys Jones^2^, Sam Hopkinson^2^, Kannan Muthuvairavan^3^, Adam Fowell^4^, Adrian Ionescu^2,4^, Emma Rees^2^

#### ^1^Swansea University, Swansea, UK; ^2^Faculty of Medicine Health and Life Sciences, Swansea University, Swansea, UK; ^3^Llwchwr Primary Care Cluster, Swansea Bay University Health Board, Swansea, UK; ^4^Swansea Bay University Health Board, Swansea, UK

*Echo Research & Practice* 2023, **10 (Suppl 1)**:ABS011

**Background:** Inappropriate use of echo wastes resources, and unnecessarily medicalises patients, potentially triggering an unnecessary cascade of investigations, with its attendant risks, expenditure and anxieties. We audited our practice to determine whether we could improve service quality by ensuring compliance with the British Society of Echocardiography (BSE) list of appropriate indications for transthoracic echo. We focused on referrals to ‘assess left ventricular (LV) function’ since this is one of the most common indications.

**Aims:** To evaluate whether:

1. Individuals triaging primary care referrals adhered to BSE-appropriate indications for echo.
2. Referrals not meeting appropriateness criteria were associated with a clinically significant result.

**Methods:** Clinical audit of 263 echo referrals from GPs (Jul 21-Feb22). Referrals were triaged by healthcare scientists and cardiologists in the local health board. Referrals with clinical evidence of disease, abnormal ECG ± abnormal NT-pro-BNP were considered appropriate. The following were considered inappropriate: minor radiographic cardiomegaly in the absence of symptoms/signs of heart failure; peripheral oedema but normal venous pressure and no evidence of cardiac disease; asymptomatic hypertension where echo would not change management. We reviewed the echo report for inappropriate referrals to determine whether there was any significant LV impairment.

**Results:** Complete information was obtained for 253 patients (57% females, mean age 63 ± 18.5y). 93 (37%) were to ‘assess LV function’ and constituted the study population. Clinical characteristics included hypertension (60%), breathlessness (54%), and peripheral oedema (33%). 69 (74%) of referrals were appropriate, 20 (22%) were inappropriate, 4 (4.3%) could not be classified. No patient in the ‘inappropriate’ group had impaired LV function but only 4(20%) were reported as normal examinations. 12 (60%) reports included abnormal LV geometry ±borderline function, 4 (20%) had mild valve disease.

**Conclusions:** A significant proportion of echo referrals to evaluate LV function were accepted in triage despite not meeting nationally accepted appropriateness criteria. These are adding to service burden and waiting times. Inappropriate referrals may produce echo findings that could be considered abnormal by GPs but may not be clinically relevant to patient presentation. We need to improve knowledge of appropriate use criteria among local GPs and those performing triage.

## ABS012 Could we offer more prudent echo services? A retrospective pilot study of level 1 echo to exclude significant disease in patients referred from primary care

### Imogen Welding^1^, Carys Jones^2^, Sam Hopkinson^2^, Kannan Muthuvairavan^3^, Adam Fowell^4^, Adrian Ionescu^2,5^, Emma Rees^2^

#### ^1^Swansea University, Swansea, UK; ^2^Faculty of Medicine Health and Life Sciences, Swansea University, Swansea, UK; ^3^Llwchwr Primary Care Cluster, Swansea Bay University Health Board, Swansea, UK; ^4^Morriston Hospital, Swansea Bay University Health Board, Swansea, UK; ^5^Swansea Bay University Health Board, Swansea, UK

*Echo Research & Practice* 2023, **10 (Suppl 1)**:ABS012

**Background:** Prudent healthcare requires us to prioritise those with greatest need, to do just what is needed—no more and no less. Anecdotal evidence from echocardiographers suggested that many referrals from GPs were to *exclude* significant disease. Level 1 echo provides a limited dataset to answer a clinical question, but it is not currently recommended for primary care. We performed a retrospective analysis of stored echo data to explore the potential utility of level 1 echo in a primary care service.

**Aims:** To identify

1. The proportion of GP referrals which may be resolved by a level 1 scan.
2. Whether an echocardiographer can accurately determine when to progress to a level 2 scan.
3. Whether significant pathology is missed when the level 1 scan is deemed sufficient.

**Methods:** A random sample of 31 patients was drawn from 263 patients referred by GPs (Jul 21-Feb22). Anonymised echo images and reports were reviewed by a Clinical Scientist (echo), before a level 1 dataset was saved to a DVD. A second echocardiographer, blind to the full dataset, analysed level 1 scans using the BSE template. No studies were excluded. Following unblinding, the echocardiographer compared level 1 with level 2 results.

**Results:** Clinical characteristics of the pilot sample were similar to the broader cohort (mean age 68, 58% female). Clinical questions were predominantly to exclude: impaired left ventricular function in patients with breathlessness 15(48%), significant valve disease in patients with a murmur 10(32%), and structural heart disease in patients with new arrhythmia 3(10%). Level 1 dataset was diagnostic in 30(97%). Level 1 scan was deemed sufficient in 21(68%) with progression to level 2 recommended in 10(32%). Of these 10, 7 were >70y with clinically significant valve disease. No clinically significant findings were missed when a level 1 scan was deemed sufficient.

**Conclusion:** Our data suggest that, to exclude LV impairment as a cause of breathlessness, a level 1 dataset interpreted by an experienced sonographer is sufficient to answer the GP’s clinical question. This approach may offer more prudent healthcare but must be confirmed in larger, prospective studies. The high prevalence of valve disease in older patients with murmurs makes this population unsuitable for level 1 scans.

## ABS014 Clinical characteristics of patients can predict echocardiographic image quality: results from the CIRI registry

### Ciaran Grafton-Clarke^1,2^, Bhalraam Uthayachandran^1,2^, Cairistine Grahame-Clark^1^, Chris Sawh^1^, Alisdair Ryding^1^, Clint Maart^1^, Omar Abdul-Samad^1^, Sunil Nair^1^, Marcus Flather^1,2^, Vassilios Vassiliou^1,2^, Pankaj Garg^1,2^

#### ^1^Norfolk and Norwich University Teaching Hospitals NHS Trust, Norwich, UK; ^2^School of Medicine, University of East Anglia, Norwich, UK

*Echo Research & Practice* 2023, **10 (Suppl 1)**:ABS014

**Background:** Echocardiography is an imaging modality that provides invaluable information in the diagnosis and treatment of disease. That said, it is a finite resource, with many healthcare services experiencing significant difficulties in service provision.

**Aims:** To determine the patient factors that are associated with poor quality or non-diagnostic echocardiography studies.

**Methods:** For all patients undergoing an echocardiogram between 2008 and 2021 at a large university teaching hospital, baseline clinical characteristics were extracted from the sonographer’s clinical report. Each variable was dichotomised and entered into a logistic regression model to determine those predictive of ‘very poor’ or ‘non-diagnostic’ images.

**Results:** 180,693 patients who underwent echocardiography between 2008 and 2021 were enrolled into the CIRI Registry (*Table 1*). Image quality was poor in 34.8% of all studies (n = 60, 6150). Four factors were predictive of poor image quality: obesity (BMI > 30 kg/m^2^; OR [odds ratio] 2.44, 95% CI [confidence interval] 2.37–2.51), inpatient scans (OR 1.96, 95% CI 1.91–2.00), tachyarrhythmias (any rate > 100 beats per minute; OR 1.17, 95% CI 1.14–1.20; p < 0.001), and male sex (OR 1.06, 95% CI 1.04–1.08) (*Figure 1a*). We developed a mosaic plot to combine these four variables to highlight the probability of poor imaging quality (*Figure 1b*), with probabilities varying from 23 to 69%.

**Conclusion:** Careful consideration of basic clinical characteristics can help determine which patients are likely to have non-diagnostic scans. This may help strained clinical services improve their diagnostic yield and develop routes to other imaging modalities in cases where echocardiography may not prove helpful. We propose a model that can be used to vet echocardiography requests to help provide a more sustainable service.

**Table 1 (abstract ABS014):** Baseline characteristics (n = 180,693) Continuous variables are presented as mean ± standard deviation. Categorical data are presented as frequencies and percentages

	Total	Probability of poor image quality	Potential modifier
**Age (years)**	63.3 ± 19.8	–	
**Gender (n, %)** Male Female	95,978 (54.0) 81,542 (46.0)	35.8% 34.0%	N/A
**Clinical setting (n, %)** Inpatient Outpatient	36,781 (20.7) 140,537 (79.3)	44.9% 32.3%	12.6% (inpatient to outpatient)
**Cardiac rhythm (n, %)** Tachyarrhythmia No tachyarrhythmia	137,043 (87.0) 20,390 (13.0)	39.4% 34.1%	5.3% (tachycardic to normocardic)
**BMI (n, %)** Underweight Normal Overweight Obese	4466 (6.0) 21,644 (28.9) 27,735 (37.0) 18,093 (24.1)	26.3% 21.5% 29.1% 49.9%	20.8% (obese to overweight)

**Figure 1 (abstract ABS014):**
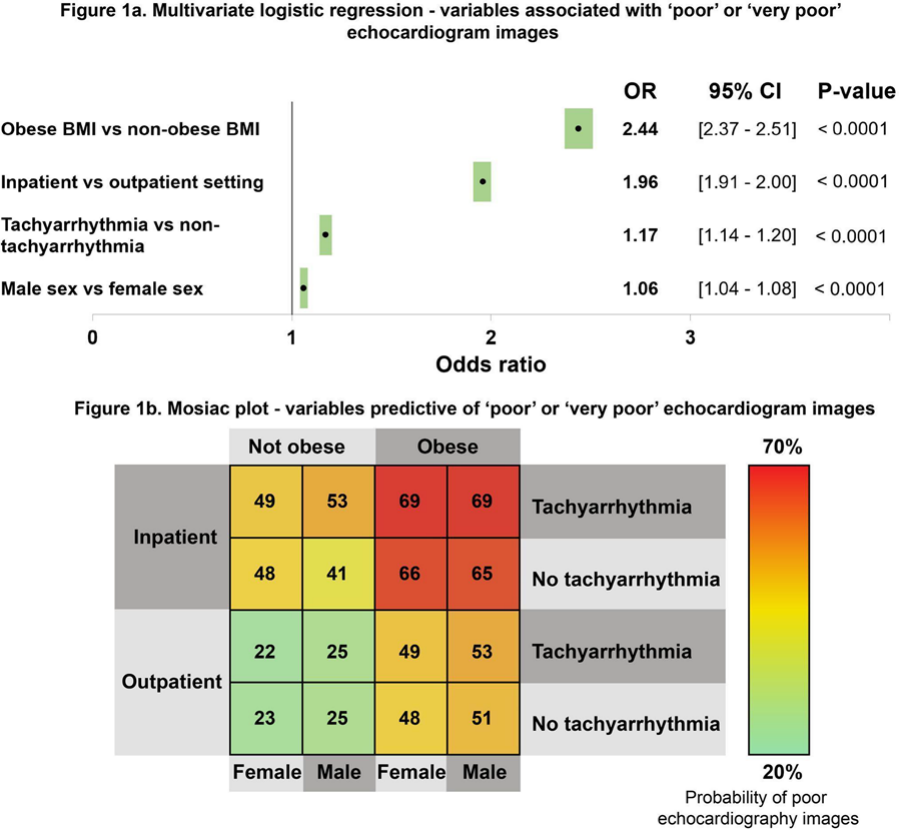
**a** Multivariate logistic regression- variables associated with 'poor' or 'very poor' echocardiogram images. **b** Mosiac plot- variables predictive of 'poor' or 'very poor' echocardiogram images

## ABS015 Feasibility of three dimensional and global longitudinal strain transthoracic echocardiography in a real-world single-centre dedicated NHS cardio-oncology clinic

### Patrick O'Driscoll^1^, Rebecca Dobson^1^

#### ^1^Liverpool Heart and Chest Hospital NHS Foundation Trust, Liverpool, UK

*Echo Research & Practice* 2023, **10 (Suppl 1)**:ABS015

**Background:** Recent publication of international cardio-oncology imaging guidance and the development of advanced analysis software has brought about a change in the routine monitoring of LV ejection fraction (LVEF) in echocardiography. Recommendations highlight 2D and 3D-LVEF and 2D-GLS as surveillance parameters of left ventricular systolic function for patients at risk of chemotherapy-related cardiac dysfunction (CTRCD).

**Aims:** We sought to determine the feasibility of the acquisition and analysis of 3D-LVEF datasets compared to 2D- GLS and 2D-LVEF within a newly developed CO service.

**Methods:** This was a single-centre retrospective analysis with minimal exclusion criteria (diagnosis of carcinoid heart disease). New patients (n = 100, 52% male, aged 67 ± 12 years) were referred to an NHS CO-TTE clinic. Using dedicated software (Philips EPIQ CVx version 7, X5-1 3D transducer and 3DQ advanced software), we sought to acquire and analyse 2D and 3D-LVEF and 2D-GLS adhering to the BSE CO-TTE protocol.

**Results:** 3D-LVEF, 2D-GLS and 2D-LVEF was achievable (n = 40, n = 73, n = 81, respectively) with no quantitative TTE imaging possible for poor TTE subjects (n = 19). There was a strong correlation between 3D LVEF and 2D LVEF (r = 0.94, mean LVEF 56.9% ± 9.8 vs 56.8% ± 9.7 respectively). Acceptable correlation was also found between 2D-GLS with 2D LVEF and 3D-LVEF (r = 0.78,) (r = 0.71, respectively). Most persistent obstacle to 3D-LVEF was poor endocardial border tracking (n = 26).

**Conclusion:** Although 3D-LVEF was found to be reliable when obtainable, acquisition and analysis was only possible in 40% of the CO-TTE cohort. Conversely, 2D-GLS has been found to be achievable, reliable and comparable to established methods of LVEF assessment in TTE, even in this difficult patient population for adequate echocardiographic windows. We are confident with more experience of 3D analysis software, feasibility of acquisition and analysis will improve.

## ABS016 Could community nurses add point-of-care ultrasound to their assessment tool kit in those with suspected heart failure?

### Sophie Moosavi^1^, Firdaus Adenwalla^2^, Annette Davis^2^, Adrian Ionescu^1,3^, Emma Rees^1^

#### ^1^Faculty of Medicine Health and Life Science, Swansea University, Swansea, UK; ^2^Neath Port Talbot Acute Clinical Team, Cimla Health and Social Care Centre, Swansea Bay University Health Board, Swansea, UK; ^3^Morriston Hospital, Swansea Bay University Health Board, Swansea, UK

*Echo Research & Practice* 2023, **10 (Suppl 1)**:ABS016

**Background:** There is evidence that with relatively brief training, point-of-care ultrasound (POCUS) by nurses is feasible and increases the diagnostic yield of the examination. The literature is dominated by accuracy studies that do not explore repeatability or implementation challenges. Despite increasing government pressure to improve diagnostic testing out-of-hospital, prior studies have focused on use by specialist nurses within hospital settings. Since non-specialist community nurses often provide initial point-of-care assessments in elderly patients at home, we sought (initially) to assess whether they could learn to detect global left ventricular systolic dysfunction (LVSD) and venous congestion accurately and reliably on ultrasound before considering potential clinical implementation.

**Methods:** Four ultrasound-novice community nurses completed a bespoke POCUS training programme comprising of a 5-day introductory course and subsequent practice period. Nurses individually analysed fourteen pre-selected focused video cases (with/without pathology) blind to clinical information. Analyses were made using a strict protocol based on qualitative assessments of specified pathology. Repeat analysis occurred two-weeks later.

**Results:** The nurses accurately (sensitivity 0.94 and specificity 0.89) and reliably detected LVSD with substantial intra-operator agreement (k =  ≥ 0.71). Abnormal IVC, pleural effusion, and B-line positivity were also detected accurately (sensitivity ≥ 0.85 and specificity ≥ 0.86) and reliably (intra-operator agreement k =  ≥ 0.71).

**Conclusion:** After dedicated training, community nurses can accurately and reliably detect LVSD and signs of venous congestion on ultrasound. Nurse-led POCUS could potentially revolutionise diagnostic community care but the complexities of implementing POCUS within this setting are unknown. Therefore, a thorough evaluation of accuracy, reliability, and clinical impact is required. We are amidst studies in this area.

## ABS017 The right assessment: utilising novel atrial markers to support pulmonary hypertension investigation

### James Willis^1^, Freya Henry^1^, Oliver Slegg^1^, Jennifer Rossdale^1^, Robert MacKenzie-Ross^1^, Jay Suntharalingam^1^, Gerry Coghlan^2^, Daniel Augustine^1,3^

#### ^1^Royal United Hospitals Bath NHS Foundation Trust, Bath, UK; ^2^Department of Cardiology, Royal Free Hospital, London, UK; ^3^Department for Health, University of Bath, Bath, UK

*Echo Research & Practice* 2023, **10 (Suppl 1)**:ABS017

**Background:** International echocardiographic guidelines for the assessment of pulmonary hypertension (PH) support a probability based approach for the presence of PH. Novel echocardiographic markers of right heart function such as strain are not included in current guidelines. These novel markers may help to detect PH.

**Aims:** To investigate the feasibility of RA reservoir strain (RArS) assessment to detect PH in patients referred for assessment to a UK shared care PH centre.

**Methods:** We retrospectively evaluated 324 patients referred for PH assessment. All patients had an echocardiogram and right heart catheter (RHC) (median interval 31 ± 30 days). PH was defined at RHC (mPAP ≥ 20 mmHg, PCWP > 15 mmHg and/or PVR ≥ 3WU). RArS was measured from the apical 4ch view. Echocardiographic measurements were made blinded to RHC data.

**Results:** RArS was measurable on 258 patients (85%) (mean strain 23.6 ± 9.7%). Those with PH had significantly lower mean RArS compared to patients with no PH (22.2 ± 9.6 vs. 27.3 ± 8.3) p < 0.001. Subgroup analysis demonstrated that post capillary PH RArS (18.6 ± 8.9) was significantly lower than pre capillary PH RArS (23.1 ± 9.8%) (p = 0.01). Pre capillary PH was lower than no PH (23.1 ± 9.8 vs. 27.3 ± 8.8%, p =  < 0.01). Comparing RArS in groups using the BSE recommended echo probability grading, Low vs High probability demonstrated a significant difference (25.7 ± 9.6 vs. 21.5 ± 9.4%) (p =  < 0.01). ROC analysis showed good sensitivity (80%) but poor specificity (50%) for cut off RArS of $\le$ 21.5% to detect PH (AUC 0.7).

**Conclusion(s):** RArS is feasible within this real-world cohort referred for PH assessment with good sensitivity. Further studies using RArS as part of a diagnostic algorithm with other markers will help to see if accuracy for detection of PH can be improved when compared with existing guidelines.

## ABS018 Pulmonary hypertension due to left heart disease: optimising echocardiographic assessment

### James Willis^1^, Freya Henry^1^, Oliver Slegg^1^, Jennifer Rossdale^1^, Robert MacKenzie-Ross^1^, Jay Suntharalingam^1^, Gerry Coghlan^2^, Daniel Augustine^1,3^

#### ^1^Royal United Hospitals Bath NHS Foundation Trust, Bath, UK; ^2^Department of Cardiology, Royal Free Hospital, London, UK; ^3^Department for Health, University of Bath, Bath, UK

*Echo Research & Practice* 2023, **10 (Suppl 1)**:ABS018

**Background:** Pulmonary hypertension (PH) secondary to left heart disease (PH-LHD) remains the most common cause of PH. Echocardiographic assessment of PH to identify those with PH-LHD is important so that prompt treatment can be started and also to try to prevent unnecessary additional tests (e.g. right heart catheterisation (RHC)). Current probability algorithms do not include specific indices for assessment of left heart disease. Novel parameters such as Left Atrial Reservoir Strain (LArS) (%) have been shown to be of benefit when assessing left heart disease.

### Aims

1. To assess the feasibility of LArS measurement in identifying those with PH-LHD
2. To identify diastolic parameters in a real-world cohort able to differentiate between precapillary PH and PH-LHD.

**Methods:** This was a retrospective evaluation of 324 patients referred for assessment of PH. All patients had an echocardiogram and RHC (median interval 31 ± 30 days). The presence of PH was defined at RHC. Diastolic parameters were calculated, and LArS was measured using a dedicated atrial strain algorithm. All echocardiographic measurements were made blinded to RHC data.

**Results:** LArS was achievable in 72% of the cohort. Precapillary, post capillary and no PH groups were arranged according to RHC data (*Table*1.0). Of all diastolic parameters, E/e’; indexed LA volume, Avg S’ and LArS were significantly worse in those with PH-LHD compared to both precapillary PH and those without PH. ROC analysis for these parameters showed acceptable ROC-AUC at 0.7–0.8, although no single parameter had both good sensitivity and specificity.

**Table 1 (abstract ABS018):** Cohort data organised into Pre-capillary PH (groups 1,3,4,5), post capillary (group 2) and No PH—diagnosed by RHC (mPAP > 20 mmHg, PCWP > 15 mmHg or PVR > 3WU) for comparison

PH Group	n	Peak E-wave velocity (cm/sec)			Peak A-wave velocity (cm/sec)			MV DT (msec)			MV E/A ratio			Mitral E/e'			LA maximum volume index (mL/BSA)			AVG Pulsed-wave TDI e' velocity (cm/sec)			AVG Pulsed-wave TDI S' velocity (cm/sec)			LAsR (%)			% with LV function > 55%
		mean		SD	mean		SD	mean		SD	mean		SD	mean		SD	mean		SD	mean		SD	mean		SD	mean		SD	
Pre-Cap PH	176	0.60	+	0.20	0.75	+	0.22	240.36	+	95.50	1.30	+	6.11	8.28	+	3.51	26.56	+	14.14	0.09	+	0.03	0.08	+	0.18	21.73	+	7.72	89%
Post Cap PH	60	1.00	+	0.39*	0.76	+	0.35	214.06	+	113.68	1.31	+	0.70	13.61	+	6.29*	46.75	+	23.64*	0.08	+	0.03	0.07	+	0.02*	15.91	+	9.21*	87%
No PH	88	0.61	+	0.20	0.69	+	0.23	237.90	+	61.29	0.90	+	0.37	7.27	+	2.56	26.69	+	13.72	0.09	+	0.03	0.08	+	0.02	24.71	+	7.39	97%
Total	324	0.68	+	0.29	0.73	+	0.25	234.83	+	91.51	1.18	+	4.60	8.86	+	4.41	30.17	+	17.81	0.09	+	0.03	0.08	+	0.02	21.50	+	8.47	-
* p = < 0.001 vs. pre-cap PH & No PH																													

**Conclusion:** LArS, in addition to E/e’, Avg S’ and indexed LA volume are able to differentiate PH-LHD from precapillary PH. LArS is feasible in this real-world cohort referred for evaluation of PH. No single diastolic marker has both good sensitivity and specificity for the detection of PH-LAD. Further work is needed to identify the optimal echocardiographic algorithm of diastolic markers to help identify PH-LHD. This could include more novel markers such as LArS and may be of additional benefit to current guidelines to help guide clinicians as to the potential presence of PH-LHD

## ABS019 Infective endocarditis echocardiography referrals: Duke’s Criteria effectiveness

### Fersha Lou Gauran^1^, Rory Wilkinson^1^, Ben McKee^1^, Fraser Kenny^1^, Khadijah Ginwalla^2^, James Willis^1^ and Daniel Augustine^1,3^

#### ^1^Royal United Hospitals Bath NHS Foundation Trust, Bath, UK; ^2^Bristol Medical School, Bristol University, Bristol, UK; ^3^Department for Health, University of Bath, Bath, UK

*Echo Research & Practice* 2023, **10 (Suppl 1)**:ABS019

**Aims:** To assess the benefit of using the modified Duke’s criteria on echocardiography referrals for infective endocarditis.

## Method:

Phase-1

46 IE TTE referrals from September–November 2019 were retrospectively analysed. Blood culture (BC) results and case-notes were reviewed for major and minor criteria to calculate a Duke’s score (Rejected/Possible/Definite). Further transesophageal echocardiography (TOE) results were reviewed.

Phase-2

114 referred for assessment of IE underwent TOE from Oct 2017-November 2019. A Duke’s score was calculated and the initial TTE referral reviewed.

**Results:** Of all of the TTE referrals made for suspicion of endocarditis, one Duke’s score was submitted as part of the referral.

Phase-1

Of the cohort, 35% (n = 16) were female (Avg-67yo) and 65% (n = 30) male (Avg-70yo). From calculation of Duke’s score: 48% rejected (n = 22), 30% possible (n = 14) and 22% definite (n = 10). 33% (n = 15) went on to have a TOE. No TOE evidence of IE found in subjects fulfilling rejected criteria. TOE results with evidence of endocarditis or unable to rule out IE (UTRO) 20% (1/5) had possible and 88% (7/8) definite (see Figure 1).

Phase-2

Of the cohort, 35% (n = 40) were female (Avg-65yo) and 65% (n = 74) were male (Avg-66yo). Calculation of Duke’s score: 32% (n = 37) rejected, 52% (n = 59) possible and 16% (n = 18) definite. Of those with TOE with evidence of / UTRO IE: 14% (n = 5) rejected, 37% (n = 22) possible and 81% (n = 13) definite (see Figure 2).

**Conclusion:** Calculating Duke’s score for endocarditis referrals as recommended in recently published BSE triage criteria is useful for risk stratifying patients. This should be included in TTE referrals for the assessment of IE.

**Figure 1 (abstract ABS019):**
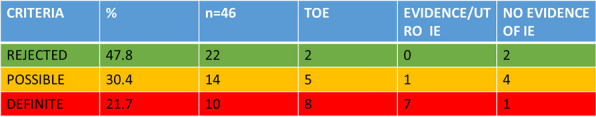
Phase 1—calculated Duke criteria for TTE referrals and the number of patients that went on to have a TOE and its results (No evidence or UTRO / Evidence of IE)

**Figure 2 (abstract ABS019):**
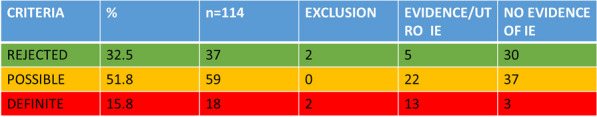
Phase 2—Duke Criteria calculated for patients that underwent a TOE and results

## ABS020 Inpatient transthoracic echocardiography service evaluation at a district general hospital: Does triaging all referrals improve waiting times for routine and urgent inpatient referrals?

### Andrea Airey^1^, Natasha Anderson^1^, Kelly Briscoe^1^, Carol Graham^1^, Natalie Hargreaves^1^, Lesley Jones^1^, Karen Pimblett^1^, Ioannis Moukas^1^

#### ^1^Warrington and Halton Hospitals NHS Foundation Trust, Warrington, UK

*Echo Research & Practice* 2023, **10 (Suppl 1)**:ABS020

**Background:** Increasing patient admissions, staff shortages and increased reliance on diagnostic tests have increased the waiting time for an inpatient transthoracic echocardiogram.

**Aims:** The purpose of the study is to ensure the service can provide diagnostic echo provision to appropriate patients within an acceptable timescale.

**Methods:** All inpatient echo referrals were retrospectively analysed at two time points: (a) November 2020–December 2020 and (b) July 2021 and August 2021. Referrals were identified from an electronic referral system and all inpatient referrals were included. Priority status indicated by referrer, date/time of referral, date/time of scan and referral indication were recorded for all referrals. Average time from referral to scan was analysed for both routine and urgent referrals. In July 2021 a triaging system was implemented utilising the 2021 British Society of Echocardiography triaging (BSE) guidelines to assess whether this improved service provision by reducing inappropriate inpatient referrals and therefore reduce inpatient waiting time.

**Results:** There were a total of 435 inpatient referrals in 2020 (a) and there was an average waiting time for a routine referral of 10 days. Urgent referrals were seen within 5 days.

In 2021 (b) there were 383 referrals for inpatient echocardiography. Average waiting time for routine referral was 52 h. All referrals for suspected endocarditis were scanned within the target timeframe of 48 h. Of all referrals 60% were prioritised as urgent by the requesting doctor. Only 10% were triaged as urgent by BSE guidelines.

**Table:** 

	Nov-Dec 2020	July–August 2021
# referrals recieved	435	383
Triaged as urgent by referrer	291 (67%)	230 (60%)
Triaged as urgent by triaging	n/a	38 (10%)
Average wait for routine referral	10 days	52 h
Average wait for urgent referral	5 days	< 24 h
% endocarditis seen within 48 h	75%	100%

**Conclusion:** Implementing the BSE 2021 triaging guidelines has a positive impact on inpatient transthoracic echo provision, reducing the number of inappropriate referrals and improving the waiting time for all referrals. Prioritisation is standardised for all referrals and therefore the right patients are getting the right tests, quicker.

## ABS021 The prevalence of valvular heart disease and left ventricular systolic dysfunction in North East London, a multi-ethnic community

### Nabila Laskar^1^, Harjit Singh^2^, Abhishek Joshi^3^, Riyaz Patel^1,4^, Sanjeev Bhattacharyya^1^, Guy Lloyd^1^

#### ^1^St Bartholomew’s Hospital, Barts Health NHS Trust, London, UK; ^2^Granville Medical Centre, Ilford, UK; ^3^Royal London Hospital, Barts Health NHS Trust, London, UK; ^4^University College London, London, UK

*Echo Research & Practice* 2023, **10 (Suppl 1)**:ABS021

**Background:** Contemporary evidence suggests clinically significant (moderate/severe) Valvular Heart Disease (VHD) affects 13.3% in people ≥ 75yrs but this data comes from rural, suburban regions with a predominant Caucasian population. We have a paucity of data of disease incidence within urban, inner city communities such as North East London (NEL), with a high migrant population, greater cardiovascular risk profile and more rheumatic heart disease. We therefore performed community based hand-held focussed echocardiography on people ≥ 65yrs old in NEL to detect left-sided VHD and left ventricular systolic dysfunction (LVSD).

**Methods:** 518 patients without known VHD or LVSD ≥ 65yrs old were scanned across 4 GP practices and 1 vaccination centre, using handheld ultrasound. An abbreviated dataset incorporating colour flow and 3/6 standard echocardiography views were used. All studies were uploaded via a secure network and read by an independent expert for verification.

**Results:** Of the 518 patients, ethnic origin was Asian 74%, White 14.4%, African 9.9% and Other 1.3%.

Newly diagnosed VHD or LVSD was found in 37% of patients. 16(6.4%) had mild LVSD, 6 (2.4%) had moderate LVSD and 2 (0.8%) had severe LVSD. Mild VHD was most common with mitral regurgitation (MR) being the most common valvular pathology (59%). Aortic Regurgitation (AR) was found in 23%, aortic stenosis (AS) in 15% and mitral stenosis (MS) in 3.5%.

**Conclusion:** The prevalence of VHD and LVSD within NEL is greater than contemporary estimates, possibly due to racial and socioeconomic factors. Handheld ultrasound is a useful tool for community detection of VHD and LVSD.

**Figure 1 (abstract ABS021):**
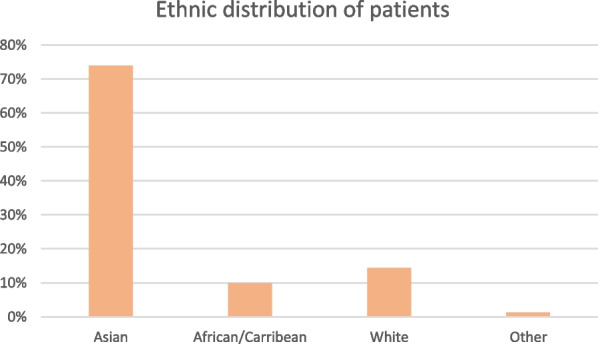
A bar chart showing the ethnic distribution of patients scanned across NEL

**Figure 2 (abstract ABS021):**
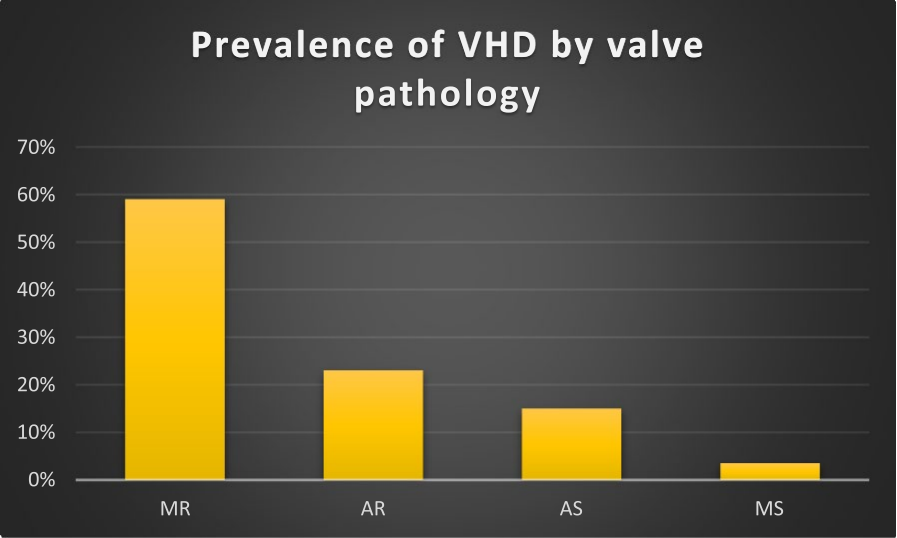
A bar chart showing incidence of VHD by pathology

**Figure 3 (abstract ABS021):**
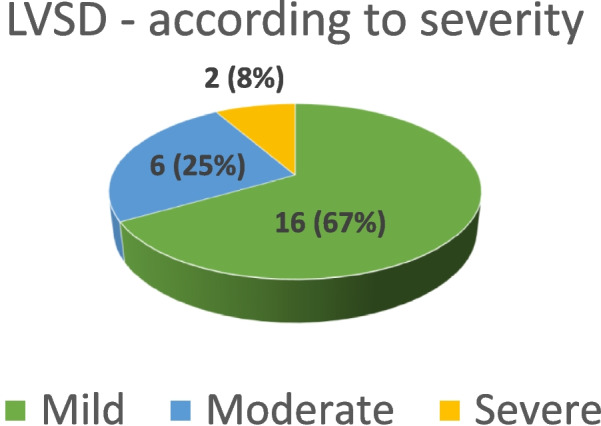
A pie chart showing prevalence of LVSD according to severity

## ABS022 Prevalence of Valvular Heart Disease in North East London—how does the prevalence differ in areas of high and low deprivation?

### Nabila Laskar^1^, Nikhil Ahluwalia^1^, Madihah Ali^2^, Harjit Singh^3^, Riyaz Patel^1,4^, Sanjeev Bhattacharyya^1^, Guy Lloyd^1^

#### ^1^St Bartholomew’s Hospital, Barts Health NHS Trust, London, UK; ^2^King's College London, London, UK; ^3^Granville Medical Centre, Ilford, UK; ^4^University College London, London, UK

*Echo Research & Practice* 2023, **10 (Suppl 1)**:ABS022

**Background:** Most of our population statistics on Valvular Heart Disease (VHD) come from rural or suburban western communities. In high migrant populations such as North East London (NEL), the burden of disease may be different relating to genetic factors, comorbidities and deprivation. Redbridge, a unique borough in NEL contains 20% of neighbourhoods amongst the most deprived in the nation and 20% of the least deprived.

We do not understand the valve disease burden in such a cohort. Planned intervention and early detection could remove 25% of valve-related heart failure hospitalisations.

**Methods:** 518 patients ≥ 65yrs old were screened for VHD using handheld echocardiography. Patients were recruited from 4 GP practices– 2 practices in a suburban sector of NEL (lower deprivation) and 2 in a more urban area (higher deprivation).

**Results:** The highest prevalence of VHD (44%) occurred in 1/2 of suburban GP practices, whilst the lowest (33%) occurred in the urban GP practice with the highest deprivation. The second suburban GP practice had a prevalence of 39%, whilst the remaining urban GP had 40%.

The mean age and proportion of female patients in the 2 practices with the highest deprivation was 74.1yrs and 49.7% compared to 72.2yrs and 55.7%. The prevalence of valve disease in the 2 practices with the highest deprivation was 36.5% compared to 41.7% (p value 0.02). The proportion of moderate/severe valve disease in the 2 practices with the highest deprivation was 22.5% compared to 25.9%.

**Conclusion:** There is a significant difference in prevalence of VHD within GP practices as little as 2 miles apart. The proportion of moderate/severe VHD is much higher than contemporary estimates but the deprivation of the area is not the explanatory factor. It is more likely that other factors especially age, ethnicity and gender explain the variance.

**Figure 1 (abstract ABS023):**
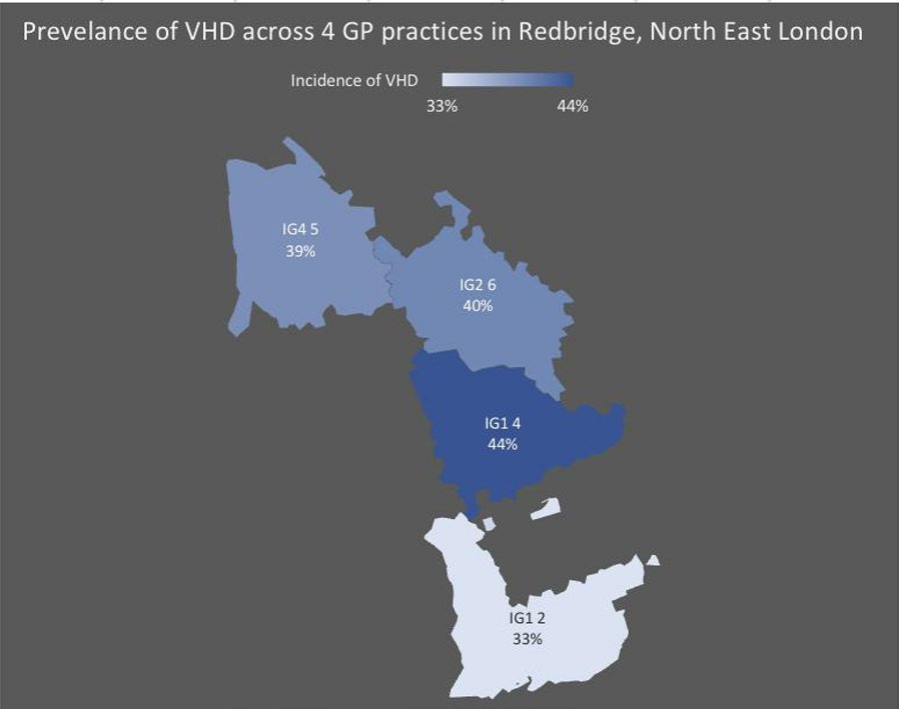
A map of Redbridge borough showing prevalence of VHD by GP practice (post code)

**Figure 2 (abstract ABS023):**
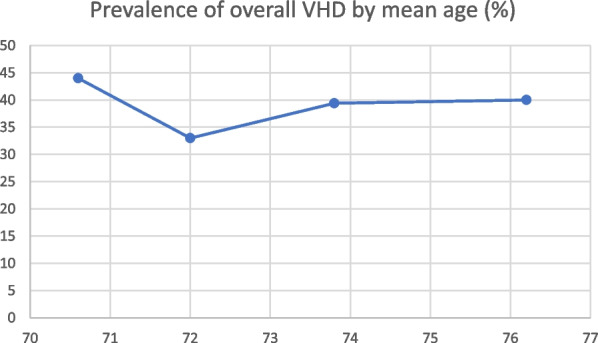
A line graph showing prevalence of VHD by mean age

**Figure 3 (abstract ABS023):**
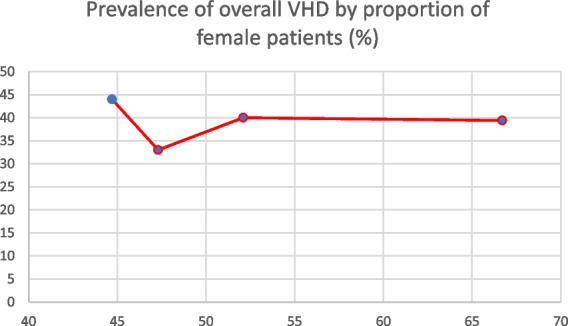
The prevalence of VHD by proportion of female patients

## ABS024 Improving triage of echocardiography requests in patients with acute stroke and acute heart failure

### Yashesh Mody^1^, Sarah Morwood^1^, James Willis^1^, Daniel Augustine^1,2^

#### ^1^Royal United Hospitals Bath, NHS Foundation Trust, Bath, UK; ^2^University of Bath, Bath, UK

*Echo Research & Practice* 2023, **10 (Suppl 1)**:ABS024

**Aim:** To audit echocardiography requests in patients with heart failure (HF) and stroke using BSE triage guidelines as a gold standard.

Methods:

- Phase 1 (audit): Retrospective review of 169 echocardiography requests in stroke patients (Jan-Dec 2021) and 40 requests in HF patients (Jan-Feb 2021). New BSE triage guidelines was used as the gold standard.
- Phase 2 (intervention): Subsequent findings were presented at the medical grand round. BSE triage guidelines were implemented and disseminated Trust wide.
- Phase 3 (re-audit): Reaudit performed over 4 weeks following implementation of BSE triage guidelines.

**Results:** Initially, 75% of stroke requests and 50% of HF requests were non-compliant with BSE triage guidelines and contained insufficient clinical information for adequate triage. Reaudit post intervention demonstrated that now 77% of referrals for stoke / HF contained sufficient information for appropriate triage. 23% of referrals were removed from the waiting list as not indicated as an inpatient.

Comparison of a 4 week period between the 2 data subsets showed that implementation of the triaging system led to 20 fewer echocardiograms being performed. Extrapolating this over 1 year would lead to a 12% reduction in inpatient echocardiography.

**Conclusion:** Implementation of BSE triage has helped to standardise the clinical information submitted on echocardiography referrals for stroke and HF. Implementation of BSE triage guidelines in our department has been shown to improve the workload within an overstretched inpatient service.

## ABS025 Quality assurance of left ventricular function assessment using transthoracic echocardiography at a tertiary centre

### Sanam Azam^1,2^, Ibrahim Sacranie^1^, Jeanelle Generoso^1^, Charlotte Fontillas^1^, Tim Husselbury^1^, James Malcolmson^1^, Sanjeev Bhattacharyya^1^, Stephen White^2^

#### ^1^Barts Health NHS Trust, London, UK; ^2^ Manchester Metropolitan University, Manchester, UK

*Echo Research & Practice* 2023, **10 (Suppl 1)**:ABS014

**Background:** Left ventricular ejection fraction (LVEF) is an important marker of systolic function assessed by transthoracic echocardiography (TTE), often predicating clinical decision making. The multiple methods to assess LVEF using TTE may yield variable results.

**Aim:** Assess image acquisition and LVEF reporting against British Society of Echocardiography (BSE) guidelines, comparing various methods of assessment.

**Methods:** Retrospective assessment of consecutive TTE studies / reports for image quality, documentation of challenging studies, LVEF (percentage & grade) and method(s) to quantify. Multi-modality imaging comparisons were performed in patients with cardiac magnetic resonance (CMR) within 6 months of TTE.

**Results:** 467 patients (56.7% male, median 59 years) were included. 150 studies (32%) reported poor endocardial definition, with a median of 5 LV segments poorly visualised. The most common method of TTE LVEF assessment was visual estimate (83%) followed by Simpson’s biplane 15%). In patients with < 2 segments poorly- visualised, LVEF assessment was performed using visual estimate in 77% and Simpson’s biplane in 23%. In patients with ≥ 2 segments poorly visualised LVEF assessment was performed using visual estimate in 90% and Simpson’s biplane in 10%.

63 patients had contemporaneous TTE + CMR with quoted LVEF %, with a median difference of 2.5% (50}13% in TTE vs 53}15% in CMR, p = 0.02). A highly significant positive correlation between the modalities was seen for both visual estimation and Simpson’s biplane method. 66 patients with TTE + CMR had graded LVEF for comparison, with 6 being discordant > 1 grade.

**Conclusion:** There is underuse of Simpson’s Biplane method regardless of number of poorly visualised segments. TTE showed good correlation with CMR irrespective of method of assessment (biplane or visual). However, TTE misclassified a proportion of patients. Routine utilisation of techniques to improve accuracy including contrast enhancing agents for poor endocardial definition and automated quantitative LVEF assessment are needed.

**Figure 1 (abstract ABS025):**
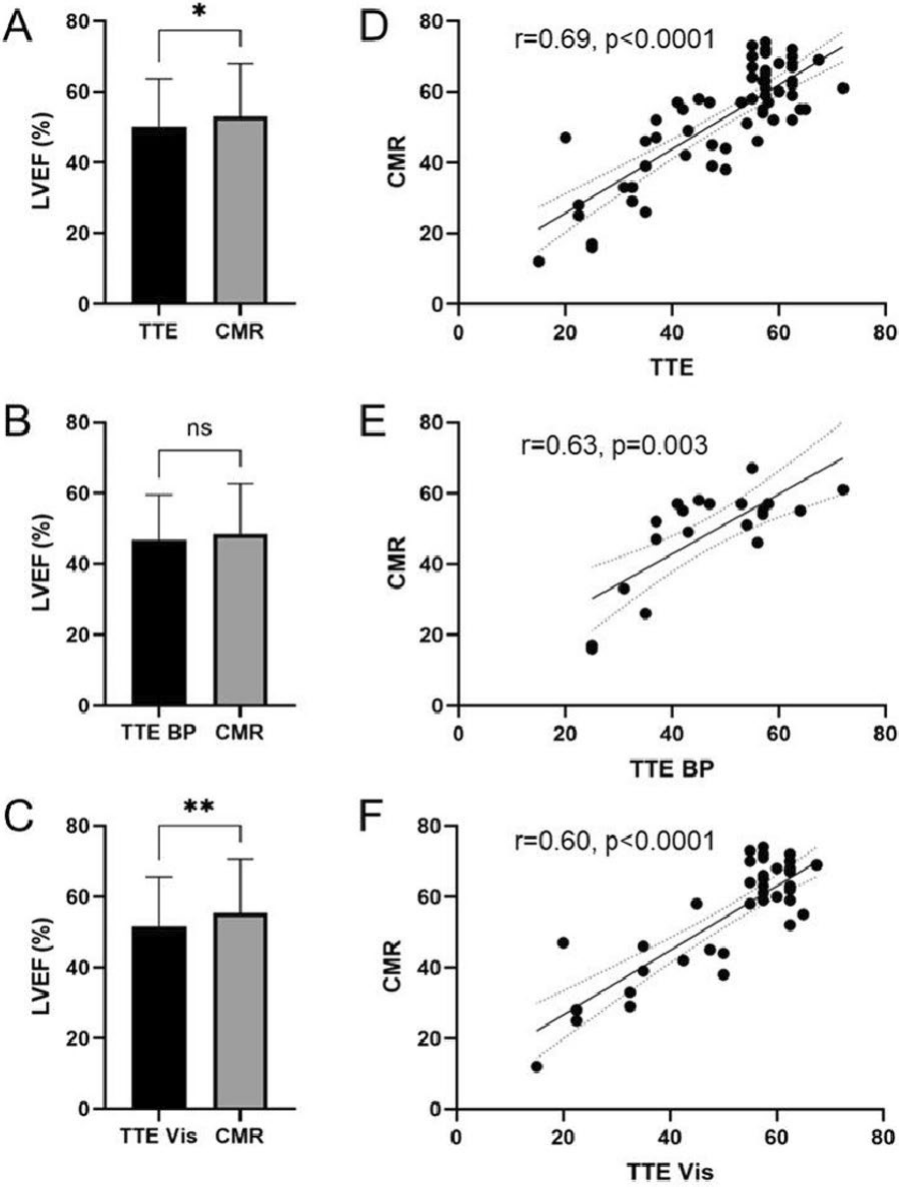
Box plots showing differences in reported LVEF across contemporaneous TTE and CMR (A), Biplane TTE and CMR (B), and visual TTE and CMR (C). Corresponding correlations with simple linear regression (Spearman r), (D, E, F respectively)

## ABS026 Hospital costs following stress echocardiography in real world settings in the UK: A costing study from the BSE-NSTEP Multi-centre study

### Casey Johnson^1^, William Woodward^1^, Annabelle McCourt^1^, Cameron Dockerill^1^, Samuel Krasner^1^, Mark Monaghan^2^, Roxy Senior^3,4^, Daniel Augustine^5^, Maria Paton^6,7^, Jamie O’Driscoll^8^, David Oxborough^9^, Keith Pierce^10^, Shaun Robinson^11^, James Willis^5^, Apostolos Tsiachristas^12^, Paul Leeson^1^, on behalf of the EVAREST/BSE-NStEP Investigators

#### ^1^Cardiovascular Clinical Research Facility, University of Oxford, Oxford, UK; ^2^Kings College Hospital NHS Foundation Trust, London, UK; ^3^Northwick Park Hospital, London North West University Healthcare NHS Trust, London, UK; ^4^Royal Brompton Hospital, Guy’s and St Thomas’ NHS Foundation Trust, London, UK; ^5^Royal United Hospitals Bath NHS Foundation Trust, Bath, UK; ^6^University of Leeds, Leeds, UK; ^7^Leeds Teaching Hospitals NHS Foundation Trust, Leeds, UK; ^8^Canterbury Christ Church University, Canterbury, UK; ^9^Liverpool Centre for Cardiovascular Science, Liverpool, UK; ^10^Manchester University NHS Foundation Trust, Manchester, UK; ^11^Imperial College Healthcare NHS Trust, London, UK; ^12^Health Economics Research Centre, Nuffield Department of Population Health, University of Oxford, Oxford, UK

Apostolos Tsiachristas and Paul Leeson are joint senior authors

*Echo Res Pract* **10**, 8 (2023). https://doi.org/10.1186/s44156-023-00020-1

*Echo Research & Practice* 2023, **10 (Suppl 1)**:ABS014

## ABS027 Clinical service evaluation of the utility of artificial intelligence based-echocardiographic quantification of left ventricular global longitudinal strain and ejection fraction in trastuzumab-treated oncology patients

### Jin Jiang^1^, Yat Wah Li^2^, Sandeep Singh Hothi^1^

#### ^1^Heart and Lung Centre, New Cross Hospital, Wolverhampton, UK; ^2^Digital Innovation Unit, New Cross Hospital, Wolverhampton, UK

*Echo Research & Practice* 2023, **10 (Suppl 1)**:ABS027

**Background:** Left ventricular (LV) global longitudinal strain (GLS) is considered a sensitive and prognostic marker in assessing ventricular systolic function in a range of diseases. Its clinical utility requires operator experience, analysis time and good image quality, and it is subject to intra- and inter-observer variability. The emergence of artificial intelligence (AI)-based analysis may offer a valuable clinical solution in measuring GLS.

**Aims:** This evaluation assesses whether AI–automated analysis has utility in assessing LV-GLS in trastuzumab-treated patients compared to manual analysis.

**Methods:** In this retrospective audit, AI-automated (Ultromic EchoGo Core) and manual (Philips QLab) measurements of GLS and LV ejection fraction (LVEF) were obtained from standard apical views in 569 transthoracic echocardiographic studies from 128 patients receiving trastuzumab.

**Results:** AI-automated analysis yielded GLS and LVEF data less frequently (12%) than conventional analysis (12 vs 33%, and 42 vs 83%, respectively). AI-automated longitudinal strain analysis in each of the apical 4-, 2-, and 3-chamber views was feasible in 51%, 47%, and 12%, respectively, compared to manually analysis (72%, 53%, 34%) (Figure 1). A fair correlation was shown between AI- and manually generated values, for both GLS (r = 0.499, P = 0.007) and LVEF (r = 0.617, P =  < 0.001) (Figures 2 & 3).

**Conclusion:** AI-automated analysis represents a potentially valuable tool for the assessment of GLS and LVEF but was successful less frequently than manual assessment. Further work should explore whether: (1) AI-based analysis is superior to less experienced humans, (2) image rejection threshold appropriateness, and (3) accuracy of AI data vs manual data.

**Figure 1 (abstract ABS027):**
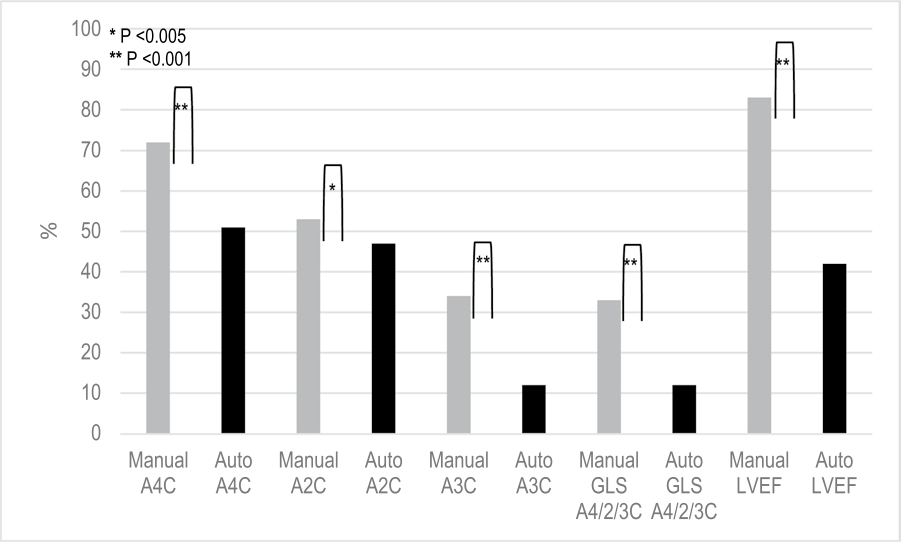
.

**Figure 2 (abstract ABS027):**
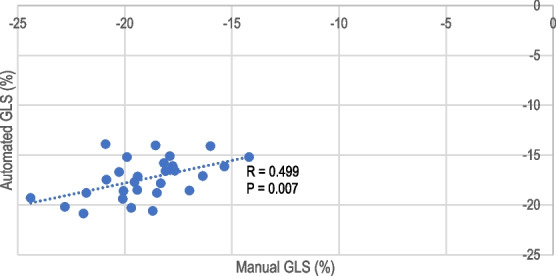
.

**Figure 3 (abstract ABS027):**
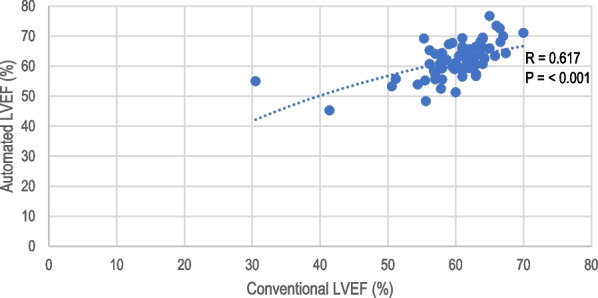
.

## ABS028 Artificial intelligence-driven segmental longitudinal strain quantification of patients with myocardial infarction

### William Hawkes^1^, Tine Deschamps^1^, Andrew Parker^1^, Gary Woodward^1^, Rizwan Sarwar^2^

#### ^1^Ultromics Ltd, Oxford, UK; ^2^University of Oxford, Oxford, UK

*Echo Research & Practice* 2023, **10 (Suppl 1)**:ABS028

**Background:** Echocardiographic imaging and quantification of segmental longitudinal strain (SLS) can provide important clinical information in patient management and risk stratification. However, high variability, poor reproducibility and the prerequisites for technical expertise limit clinical use and applicability. Automated quantification of SLS, driven by AI, has the potential to overcome these limitations and quantify myocardial function unbiased.

**Methods:** Using EchoGo Core v2.0, we retrospectively analysed a publicly available dataset (Kaggle.com, HMC-QU Dataset) of 161 apical 4 chamber transthoracic echocardiograms from patients admitted to Hamad Medical Corporation with acute ST-elevation myocardial infarction (MI) between 2018–2019. Infarcted segments were identified by full agreement between 3 cardiologists.

T-tests were used to assess the differences in peak SLS between infarcted and non-infarcted segments. Prediction of infarct presence and severity using peak SLS is analyzed using receiver operator characteristics (ROC) curves. The correlation with mean peak SLS and total number of infarcts over all segments was assessed using ANOVA and subsequent t-test with Benjamini–Hochberg correction. ROC analysis was performed to assess prediction of infarct presence (0 vs 1–6 infarcts). Correlation between peak SLS in the 6 segments was, irrespective of infarct presence, assessed through paired Pearson correlation tests.

**Results:** MI of individual segments was present in 93 patients. Infarcted segments were predominantly in the apical Inferoseptum and neighboring 2 segments. Mean peak SLS significantly increased with infarct presence in each individual segment. ROC curves stratifying infarct presence by peak SLS achieved area under the curve (AUC) values between 0.727 and 0.825. Mean peak SLS over all segments significantly increased with increasing number of infarcts (p < 0.001). ROC curves show an increase in prediction accuracy with increasing infarct severity, with AUC = 0.681 for 0 vs 1 infarcts, going to > 0.9 for 0 vs 3–6 segments. Peak SLS is significantly correlated between all segments (p < 0.001), with the strongest correlations between neighboring segments (R > 0.69).

**Conclusion:** Unbiased assessment of infarct presence and severity is feasible with automated AI quantification of peak SLS. Regional systolic function was measured to deteriorate with both the presence and severity of infarcts and was strongly correlated with that of neighboring segments, indicating that the automated analysis was sensitive to concomitant dysfunction in non-infarcted segments.

**Figure 1 (abstract ABS028):**
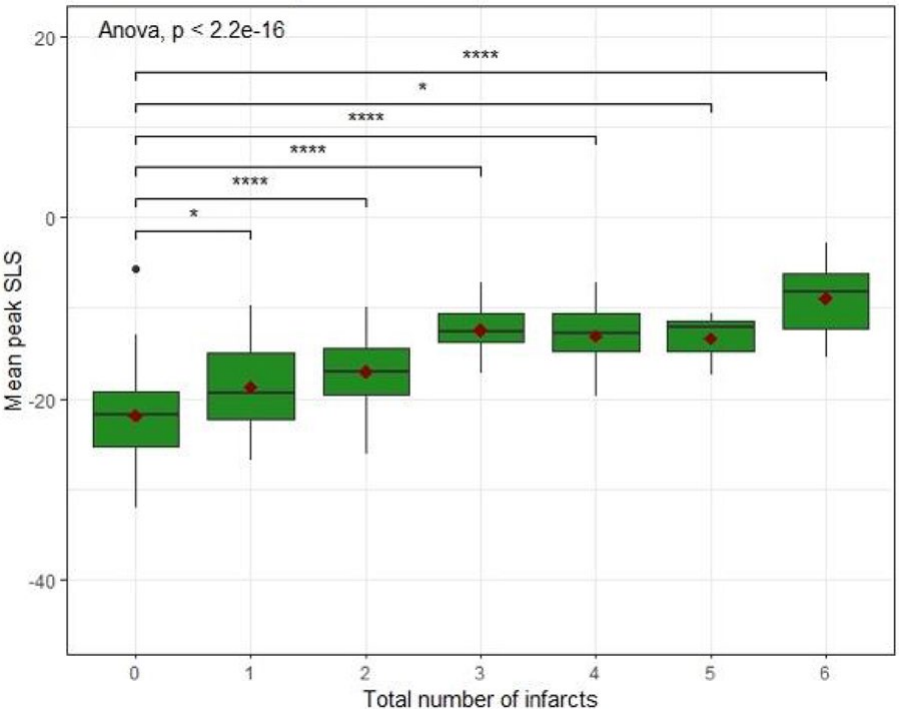
Mean Peak SLS by number of infarcted segments

**Figure 2 (abstract ABS028):**
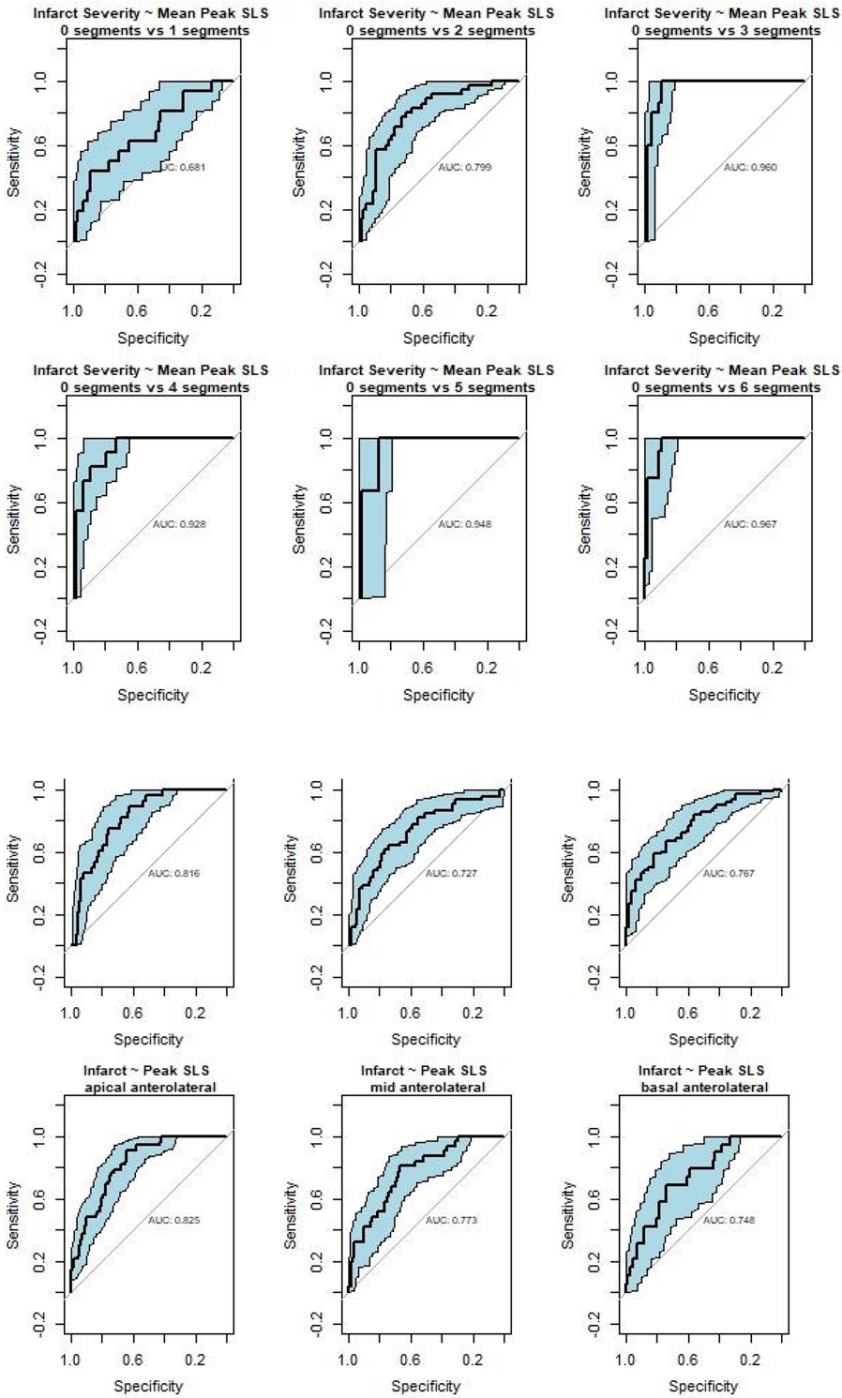
Receiver operating characteristic curves for prediction of infarct severity using mean peak SLS

## ABS029 What are the barriers to implementing global longitudinal strain in routine practice?

### Emma Lane^1^, Lindsey Fry^1^, Paul R Kalra^1^, Simon Kolstoe^2^

#### ^1^Portsmouth Hospitals University NHS Trust, Portsmouth, UK; ^2^University of Portsmouth Portsmouth, UK

*Echo Research & Practice* 2023, **10 (Suppl 1)**:ABS029

**Introduction:** There is growing evidence that global longitudinal stain (GLS) is an early predictor of left ventricular systolic dysfunction. However, it is not performed routinely in clinical practice in most echocardiography departments. Introducing new techniques into clinical practice can present challenges at the individual, departmental and societal level and understanding potential barriers is fundamental to facilitate successful implementation.

**Purpose:** To identify potential barriers to the use of GLS in clinical practice in England.

**Methods:** Online and anonymous questionnaire, adapted from determinant of behaviour questionnaire (Huijg *et al.*, 2014), to BSE physiologists/scientists within England to assess current practice and barriers prior to BSE cardio-oncology guidelines 2021. Demographic data, individual experience, departmental practice, opinions on GLS and determinants of behaviours were recorded using Likert scales and free text responses.

**Results:** A total of 55 responses were received. A majority agreed that GLS can improve patient care (85.2%) however less than half are performing GLS regularly (48.1%). Emerging themes for barriers to use included: time for including GLS into appointment time, lack of training and suboptimal image quality.

**Figure 1 (abstract ABS029):**
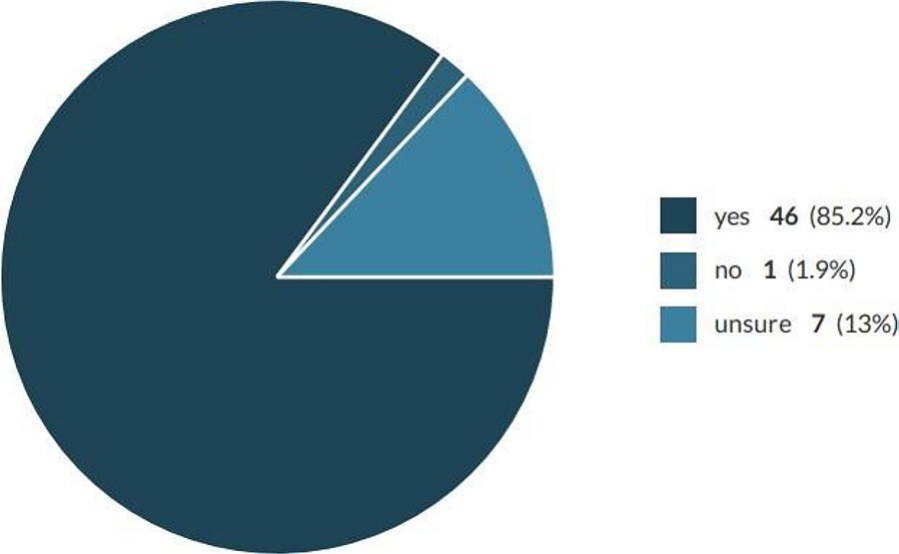
Do you agree with the following: GLS can improve clinical decision making which optimises individualised care

**Figure 2 (abstract ABS029):**
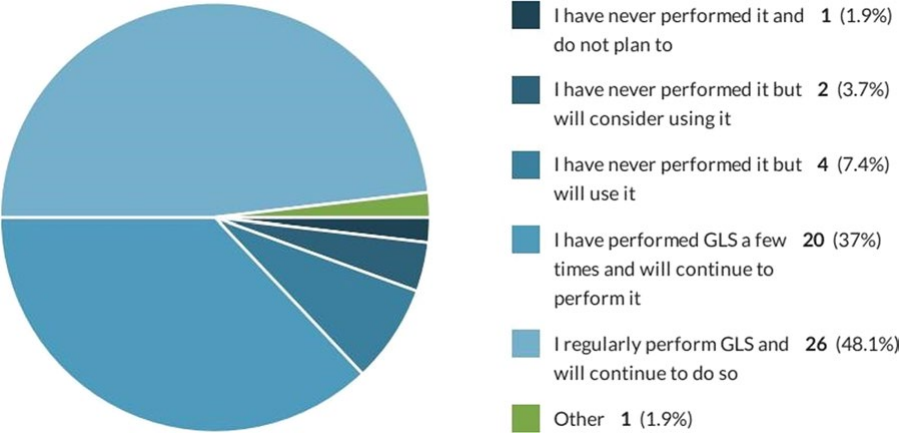
How often do you use GLS for LV assessment

**Figure 3 (abstract ABS029):**
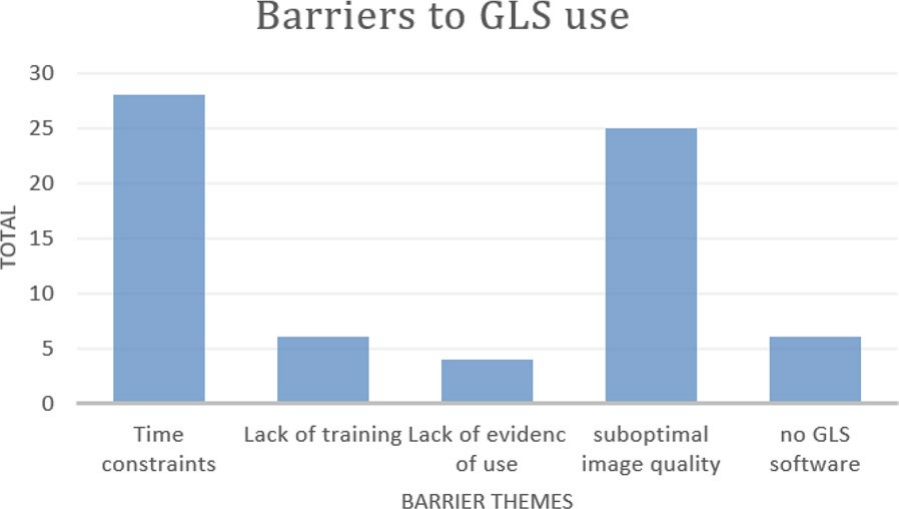
Reported barriers to using GLS

## Conclusions

The important use of GLS in cardio-oncology is highlighted in the 2021 BSE guidelines. The questionnaire identified key areas that need development plans to ensure GLS in incorporated into practice:

- Training at individual, departmental and national level on performing GLS
- Planned training clinics with time to perform GLS and for image optimisation for GLS calculations.

## ABS031 Impact of geographical origin upon left ventricular remodelling in the black paediatric male athletes’ heart

### Gavin McClean^1,2,3^, Mathew G Wilson^2,3,4^, Guido Pieles^4,5,7^, Victoria Watt^6,7^, Carmen Adamuz^7^, Keith P George^3^, Nathan R Riding^2,4^, David Oxborough^3^

#### ^1^St Bartholomew’s Hospital, Barts Health NHS Trust, London, UK; ^2^Athlete Health and Performance Research Centre, Aspetar Orthopaedic and Sports Medicine Hospital, Qatar; ^3^Research Institute for Sport and Exercise Science, Liverpool John Moores University, UK; ^4^Institute of Sport Exercise and Health (ISEH), University College London, London, UK; ^5^National Institute for Health Research (NIHR) Cardiovascular Biomedical Research Centre, Congenital Heart Unit, Bristol Royal Hospital for Children and Bristol Heart Institute, Bristol, UK; ^6^Wythenshawe Hospital, Manchester University NHS Foundation Trust, Manchester, UK; ^7^Department of Sports Medicine, Aspetar Orthopaedic and Sports Medicine Hospital, Qatar

*Echo Research & Practice* 2023, **10 (Suppl 1)**:ABS031

**Background:** Until recently, little attention has been paid to whether the heart of the black athlete is universal, or whether substantial differences exist according to geographic origin. Whilst West African athletes have been shown to demonstrate more pronounced left ventricular remodelling than West Asian and East African counterparts, after accounting for body size. The relative impact of geographical origin upon the paediatric athletes’ heart remains unknown. We examined the impact of geographical origin upon left ventricular (LV) structural remodelling in the black paediatric athletes’ heart.

**Methods:** 200 (88 West Asian, 82 West African and 60 East African) male paediatric (11–18 years) athletes, were evaluated by 2D echocardiography as per British Society of Echocardiography recommendations, with geographical region categorised by United Nations definitions (Table1; Figure 1). LV dilatation and hypertrophy were categorised by the Paediatric Heart Network Z-Scores.

**Results:** Paediatric West African athletes presented significantly greater intraventricular septal wall thickness during diastole than East African and West Asian athletes, respectively (Table 2). East African athletes presented significantly smaller posterior wall thickness during diastole than West Asian and African athletes, respectively. Upon application of the Paediatric Heart Network Z-Scores to account for body size, statistical differences in the prevalence of hypertrophy did not differ by geographical origin.

**Conclusion:** In contrast to findings in the adult male black athletes’ heart, differences in the prevalence of left ventricular hypertrophy were removed after accounting for BSA. Findings likely attributable to reduced years of training and incomplete pubertal development.

**Table 1 (abstract ABS031):** Anthropometric data of black paediatric athletes by geographical origin

	West Asian (n = 88)	West African (n = 52)	East African (n = 60)
Height, cm	170.0 ± 9.0^a^	177.8 ± 13.0^b,c^	168.9 ± 12.0^c^
Weight, kg	64.1 ± 16.6^b^	64.5 ± 15.7^b^	55.4 ± 12.2^a,c^
BSA	1.73 ± 0.26^b^	1.77 ± 0.13^b^	1.60 ± 0.23^a,c^
Age, years	15.4 ± 1.9	15.0 ± 2.0	15.5 ± 2.1

Cm, centimeters; kg, kilograms; n, number of participants

^a^Statistical difference vs. West African

^b^Statistical difference vs. East African

^c^Statistical difference vs. West Asian

**Table 2 (abstract ABS031):** Left ventricle size of black paediatric athletes by geographical origin, with application of Lopez et al^1^ Z-scores

	West Asian (n = 88)	West African (n = 52)	East African (n = 60)
LVIDd, mm	48.5 ± 4.2	49.1 ± 5.4	47.9 ± 4.7
LVIDd ≥ 2Z n(%)	1 (1.1)	1 (1.9)	0 (0)
IVSd, mm	7.7 ± 1.3^a^	8.2 ± 1.2^b,c^	7.5 ± 1.3^a^
IVSD ≥ 2Z n(%)	4 (45.5)	5 (9.6)	2 (3.3)
PWTd, mm	7.9 ± 1.3^b^	8.1 ± 1.2^b^	7.4 ± 1.3^a,c^
PWTd ≥ 2Z n(%)	5 (5.7)	5 (9.6)	3 (5)
RWT	0.32 ± 0.05	0.33 ± 0.06	0.31 ± 0.05

LVIDd, left ventricle Internal diameter; IVSd, intraventricular septal wall thickness, and; PWTd, Posterior Wall Thickness during diastole; mm, millimetres; n, number of participants

^a^Statistical difference vs. West African

^b^Statistical difference vs. East African

^c^Statistical difference vs. West Asian

**Figure 1 (abstract ABS031):**
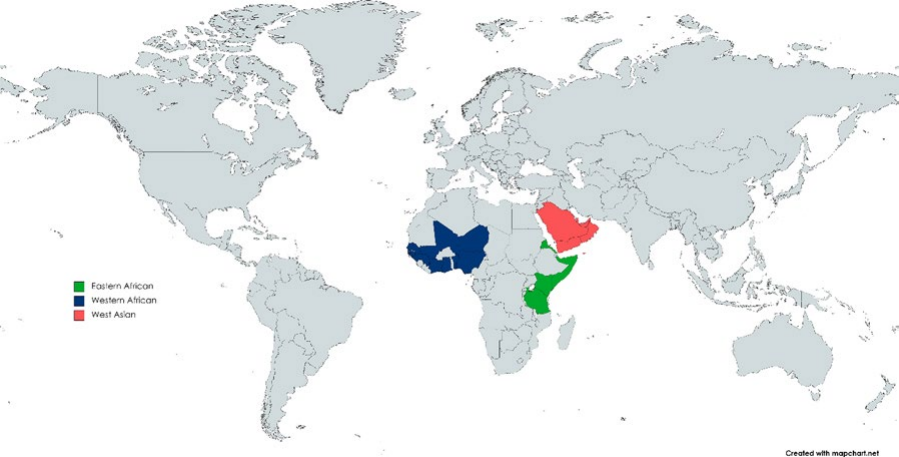
Global representation of black paediatric athletes included within the study as per geographical region

## ABS032 LVEF measured with same day echocardiography and CMR in patients with suspected cardiotoxicity

### Muhummad Sohaib Nazir^1,2^, Joseph Okafor^3^, Theodore Murphy^1^, Maria Sol Andres^1^, Sivatharshini Ramalingham^1^, Stuart D Rosen^1,5^, Amedeo Chiribiri^2^, Sven Plein^2^, Sanjay Prasad^4,5^, Raad Mohiaddin^4,5^, Dudley J Pennell^4,5^, A John Baksi^4,5^, Rajdeep Khattar^3^, Alexander R Lyon^1,5^

#### ^1^Cardio-Oncology Service, Royal Brompton and Harefield Hospitals, Guy’s and St Thomas’ NHS Foundation Trust, London, UK; ^2^School of Biomedical Engineering and Imaging Sciences, King’s College London, Guy’s and St Thomas’ Hospital, London, UK; ^3^Department of Echocardiography, Royal Brompton and Harefield Hospitals, Guy’s and St Thomas’ NHS Foundation Trust, London, UK; ^4^Cardiovascular Magnetic Resonance Unit, Royal Brompton and Harefield Hospitals, Guy’s and St Thomas’ NHS Foundation Trust, London, UK; ^5^National Heart and Lung Institute, Imperial College London, London, UK

*Echo Research & Practice* 2023, **10 (Suppl 1)**:ABS032

**Background:** LVEF is used for assessment of cancer therapy-related cardiac dysfunction (CTRCD) in patients who receive cancer therapies. Cardiovascular Magnetic Resonance (CMR) is the reference standard for LVEF assessment, but echocardiography is most widely used. This study sought to compare LVEF measured by echocardiography and CMR in cancer patients with suspected cardiotoxicity and assess potential impact on clinical decision-making.

**Methods:** In this prospective single-center observational study, 745 patients underwent same-day imaging with echocardiography and CMR. A sub-set of 74 patients also had 3D echocardiography-derived LVEF. Agreement of LVEF was determined by Bland–Altman analysis.

**Results:** Mean age 60 ± 5 years, 62% female. LVEF measured by 2D echocardiography was significantly lower compared to CMR, (median 60% [interquartile range 54–65%]) vs 63% [interquartile range 56–69%], p < 0.001). Using Bland–Altman analysis, mean bias was -3.7 ± 7.6% (95% limits of agreement [LOA] -18.5 to 11.1%) of 2D echocardiography versus CMR derived LVEF (Figure 1). Among 74 patients in whom CMR, 3D echocardiography and 2D echocardiography were performed, LVEF was 60.0 ± 10.4%, 58.4 ± 9.4% and 57.2 ± 8.9%, respectively (p = 0.0006) (Figure 2). There was better agreement with 3D echocardiography and CMR derived LVEF (mean bias -1.6 ± 6.3 [95% LOA -13.9 to 10.7%]) compared to 2D echocardiography and CMR derived LVEF (mean bias of -2.8 ± 6.3 [95% LOA-15.2 to 9.6%]), (p = 0.02).

**Conclusion:** 2D echocardiography and CMR derived LVEF measurements are not interchangeable. 2D echocardiography has variations of up to ± 15% compared to CMR. 3D echocardiography has better agreement with CMR derived LVEF. This study supports the use of 3D echocardiography in cardio-oncology patients and suspected CTRCD.

**Figure 1 (abstract ABS032):**
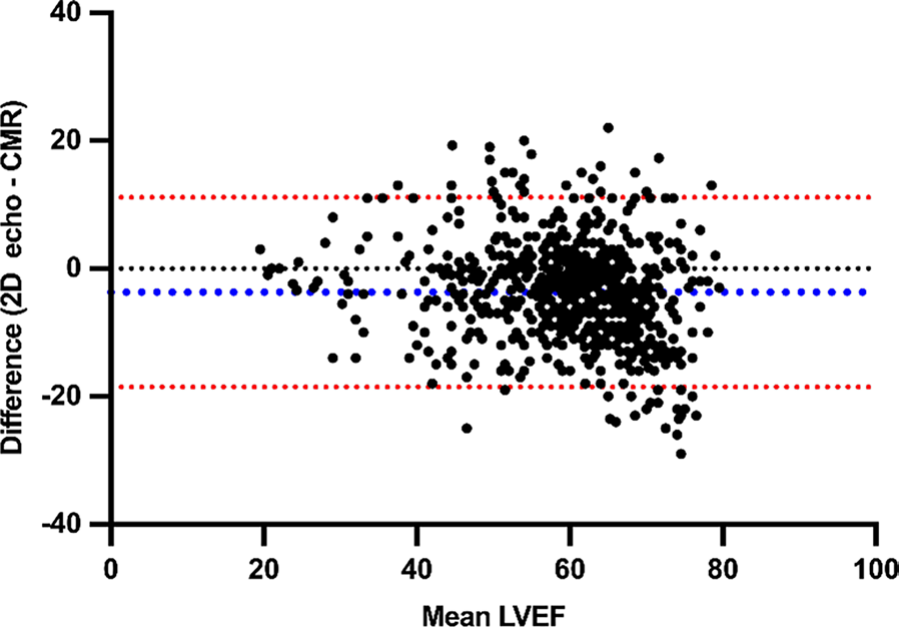
Bland–Altman analysis of same day left ventricular ejection fraction (LVEF) with 2D echocardiography and Cardiovascular Magnetic Resonance (CMR) on the whole cohort (n = 745). Dashed blue line: mean bias. Dashed red lines: 95% upper and lower limits of agreements

**Figure 2 (abstract ABS032):**
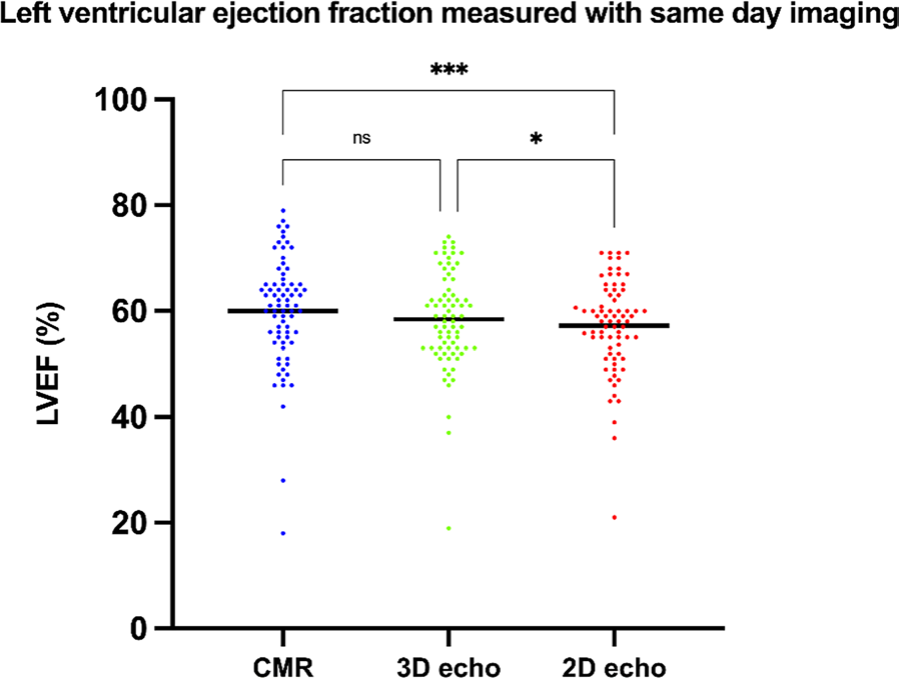
Left ventricular ejection measurements for CMR, 3D echocardiography and 2D echocardiography in the predefined substudy (n = 74)

## ABS033 Altered torsion mechanics on exercise in HCM: does it matter?

### Laszlo Halmai^1^, Attila Kardos^1^, Jose Samoes^1^, Attila Nemes^2^, Antonis Pantazis^3^

#### ^1^Department of Cardiology, Milton Keynes University Hospital NHS Trust, Milton Keynes, UK; ^2^Department of Medicine and Cardiology Centre, University of Szeged, Szeged Hungary; ^3^ICC Services and Cardiomyopathy Unit, Royal Brompton and Harefield Hospitals, Guy’s and St Thomas’ NHS Foundation Trust, London, UK

*Echo Research & Practice* 2023, **10 (Suppl 1)**:ABS033

**Background:** Hypertrophic obstructive cardiomyopathy (HOCM) is characterized by muscle hypertrophy and fibrosis, interfering with force generation and relaxation. Abnormal ventricular (LV) myocardial deformation have been demonstrated in patients with HOCM at rest, but there is lack of data regarding the deformational mechanics in exercise.

**Aims:** We wanted to assess the adaptability of LV deformational behaviour to physical exercise in HCM patients as compared to healthy control subjects.

**Methods:** 24 obstructive HOCM (age 51.2 ± 14.2yrs; 16 men, LVOT-obstruction 56 ± 19 mmHg) and 32 control subjects (50.9 ± 6.8 yrs, 19 men from the MAGYAR-PATH Registry) underwent supine bicycle stress echocardiography (ESE) with measurements of 2D- and Doppler, 2D-Speckle Tracking Tracking Imaging and 3D-Full Volume Analysis. Conventional LV and RV functional measurements; longitudinal (LS), circumferential (CS) values; peak Twist and Torsion angles; post-systolic shortening index (PSS) and diastolic phase indices (Untwisting time—UTT and rate—UTR); LV/RV-EF by 3D-Full Volume Analysis was performed off-line by TomTec Arena™ software package both at rest and on submaximal ESE.

**Results:** The HOCM group had lower resting LS (-14.6 ± 4.5 vs 18.4 ± 2.6%, p < 001) but higher CS (-32.9 ± 5.1 vs 28.8 ± 2.3%, p < 0.001) and Twist angles (9.9 ± 2.6 vs 6.1 ± 2.2º, p < 0.01) than control subjects. Exercise induced an increase in all strains in control subjects, but much less in HOCM (LS: -21.4 ± 3.5 vs 15.1 ± 3.0% and CS: -33.9 ± 3.6 vs 34.1 ± 4.2% in HOCM, p < 0.02 in controls, NS in HOCM); the increase of Twist angle was minimal in HOCM (Δ1.2 ± 1.2 vs Δ3.6 ± 2.3º in controls, p < 0.01). The PSS was more pronounced on ESE in HOCM than in controls (46.6 ± 12 vs 21.2 ± 9.6% in controls, p < 0.001). Peak UTR was slower (118 ± 2.1 vs 133.1 ± 14.1 º/s) during ESE and occured later (141 ± 19 vs 121 ± 9.1% of systolic time, p < 0.02) in HOCM than in controls. There was significant relationship between Twist and UTR in control subjects (β = -0.0807, p < 0.001), but not in HOCM (β = -0.0046, p = 0.05).

**Conclusions:** The HOCM patients had significantly impaired strain-adaptability; developed post-systolic shortening and no LV Torsional reserve was found on exercise. Decreased and delayed UTR indices were also detected, becoming more pronounced in the HOCM group. These findings support evidence for reduced systolic-diastolic coupling efficiency, assessed by Twist-Untwist mechanics in HOCM patients, which can contribute to the development of exercise-related symptoms and the dynamic LVOT-obstruction. This unique pattern of deformational behaviour to exercise can help in the differential diagnostic workup in patients with LV hypertrophy of unknown aetiology and also would hold additional value in the risk stratification process for patients with different cardiomyopathies.

